# Perspectives of women and partners from migrant and refugee backgrounds accessing the Cross Cultural Worker Service in maternity and early childhood services—a survey study

**DOI:** 10.1186/s12913-023-10194-3

**Published:** 2023-11-10

**Authors:** Helen J. Rogers, Caroline S. E. Homer AO, Amanda Henry

**Affiliations:** 1 Child, Youth & Family Services, South Eastern Sydney Local Health District, Sydney, NSW 2010 Australia; 2 Discipline of Women’s Health, School of Clinical Medicine, University of NSW (UNSW), Sydney, NSW 2000 Australia; 3 Maternal and Child Health, Burnet Institute, Melbourne, Vic 3004 Australia; 4 Centre for Midwifery and Child and Family Health, Faculty of Health, University of Technology Sydney), Sydney, NSW 2007 Australia; 5 Department of Women’s and Children’s Health, St George Hospital, Sydney, NSW 2217 Australia; 6 Australia Global Women’s Health Program, The George Institute for Global Health, Sydney, NSW 2042 Australia

**Keywords:** Health equity, Culturally responsive, Perinatal, Migrant, Refugee, Bicultural worker, Navigation support, Fathers, Parents, Partners

## Abstract

**Background:**

Women from migrant and refugee backgrounds living in high-income countries have increased risk of adverse perinatal outcomes and report lower satisfaction with perinatal healthcare. In Sydney, Australia, a new service known as the Cross Cultural Workers (CCWs) in Maternity and Child and Family Health Service (the CCW Service) was implemented to support such women and families from pregnancy to the early parenting period. This study aimed to ascertain the experiences of women and their partners engaging with the CCW Service.

**Methods:**

A survey study was undertaken. Women accessing the CCW Service were recruited during pregnancy and were asked to complete surveys at three time points: in the third trimester of pregnancy, at 6 and 12 months postpartum. Their partners were invited to complete a survey at 6 months postpartum. Survey data were analysed to compare satisfaction, usefulness, number of CCW interactions, cultural sensitivity, and service improvement recommendations across all three survey timepoints.

**Results:**

A total of 231 surveys were received: 113 during pregnancy, 50 at 6-months postpartum, 44 at 12-months postpartum, and 24 partner surveys. Participants in all surveys reported the CCW Service to be useful (84–94%), stating that it increased their understanding of pregnancy, birth and parenting (95–100%), and that they would recommend the CCW Service (92–98%). Participants experienced a high level of satisfaction (88–95%) irrespective of the number of CCW interactions (*p* = 0.42). Thoughts on becoming a mother or parent were more positive after meeting the CCW than before for both women (*p* = 0.01) and partners (*p* = 0.12). Suggestions for CCW Service improvement were to 1) increase the provision of information, specifically financial entitlements, postnatal depression, and support services, 2) increase involvement of partners in care, 3) increase the CCW workforce/or number of CCWs.

**Conclusion:**

The CCW Service was associated with positive experiences and high rates of satisfaction at all timepoints. This service has the potential to inform the implementation of similar models of care that improve accessibility, the perinatal experience, and respond to the unique needs of women and families from migrant and refugee backgrounds.

**Supplementary Information:**

The online version contains supplementary material available at 10.1186/s12913-023-10194-3.

## Background

Globally, the scale of international migration is increasing [1]. In 2020, an estimated 281 million people, 3.6% of the global population, were living in a country other than their country of birth, an increase from 221 million people (3.2%) since 2010 [1]. Approximately 48% of migrants are women, many of whom are of childbearing age [2].Their experiences after migration are key determinants of health and well-being [3]. The settlement experience for women may be challenged by the loss of the familial and social support structures in their home countries, and pre-migration trauma, which have significant impact on their mental health and wellbeing [4, 5].

Women from migrant and refugee backgrounds settling in high-income countries (HIC) have increased adverse perinatal outcomes compared to women born in the host country. This includes, increased rates of mental health issues [6–10], lower levels of attendance [10], and later access to antenatal care [11–13], increased congenital anomalies and preterm birth [3, 8], and higher rates of smaller for gestational age infants [14–16], stillbirth [16–20], and infant admission to neonatal care [21, 22]. These adverse perinatal outcomes may have a profound impact on a child’s development, and lifelong health outcomes [23, 24].

Women and families from migrant and refugee backgrounds experience inequities accessing maternal and child health services [3, 25, 26], including limited health literacy, language barriers, and cost [27, 28]. Women have described dissatisfaction with maternity care due to service providers limited cultural knowledge, stereotypical assumptions, limited availability of interpreters, and the healthcare system not being culturally responsive [29, 30]. Additionally, for newly arrived families, the demands of settlement, including housing and employment, may be prioritised over accessing maternity and child health services. Legal frameworks, politics and policies of host countries also define many of the rights, cost, social opportunities and access to healthcare [31, 32].

In the Australian context, Medicare is Australia’s publicly-funded universal health insurance scheme. Access to publicly-funded maternal health services is free to women who are refugees, seeking asylum, Australian citizens, permanent residents, and New Zealand citizens. The Australian Government recommends all temporary visa holders take out private health insurance, and private health insurance is a prerequisite for some temporary visa types. However, the level of benefit available may vary between insurance providers, and a 12-month waiting period for pregnancy-related care may be applied [33].

The importance of services responding to improve perinatal outcomes, the experience of care, and overcome barriers to access, is a recognised public health priority [3, 8, 25, 34]. However, implementation and evaluation of targeted models of care to meet the needs of women and families from migrant and refugee backgrounds are limited [35–37]. A systematic scoping review of pregnancy and postpartum models of care for women from migrant and refugee background living in high income countries (HIC) mapped effectiveness in terms of improving maternal and infant health outcomes, acceptability and appropriateness from the perspective of women and service providers [38]. A diverse range of potentially effective models were identified, including female paraprofessional bicultural/bilingual workers [39–49], multidisciplinary group models of antenatal care [39, 50], and specialised antenatal clinics [5, 51, 52]. Key elements included culturally responsive continuity of care, effective communication, psychosocial and practical support, and support to navigate systems. The review highlighted the need to demonstrate effectiveness and acceptability from multiple perspectives including experiences of women, their partners, and service providers [38].

The Cross-Cultural Workers (CCWs) in Maternity and Child and Family Health Services (the CCW Service) was implemented during 2017 in metropolitan Sydney, Australia. The service supports women and families from migrant, refugee, and asylum seeker backgrounds to access and navigate maternity, child and family health (CFH) services, and community-based organisations through pregnancy, and into early childhood until school entry (5 years). This study aims to 1) describe women and their partners experience engaging with the CCW Service, and 2) to examine and compare women’s experience with the CCW Service across the three survey timepoints; in the third trimester of pregnancy, at 6 and 12 months postpartum.

## Methods

### Design

A survey study was undertaken using online and paper-based surveys.

### Setting

The CCW Service is based in South-Eastern Sydney, Australia, a culturally diverse metropolitan area. There are three publicly-funded maternity facilities with approximately 10,000 births per year total, with 38% of women being born in a non-English speaking country (Glassick S and Rogers HJ. ObstetriX Data Analysis Project Report: A descriptive analysis of women birthing in South Eastern Sydney Local Health District 1 July 2012 to 30 June 2015. 2017: unpublished). Public antenatal care is offered through midwifery-led and doctor-led clinics, midwifery group practice, group models of antenatal care, or shared care arrangements between a general practitioner (family doctor) and hospital antenatal clinics. Following birth and discharge, women and families have access to universal child and family health nursing services provided from birth until school entry.

### The Cross Cultural Worker Service model of care

The CCW Service is an enhanced model of care provided alongside existing maternity and CFH services. Initial funding for the model was provided by the CFH Directorate within the Local Health District until 2022. Following a successful business case/demonstration of value of the Service, the Local Health District now provides on going funding. The Service employs three female, part-time, CCWs who have lived experience of the migration journey, and are fluent in both their country-of-origin language and English. This includes CCWs from Nepal (Nepali and Hindi speaking), Bangladesh (Bangla, Hindi and Urdu speaking) and Indonesia (Bahasa speaking). Incidentally, women from these backgrounds were identified as being at highest need in an internal review prior to service set-up.

**Fig. 1:**
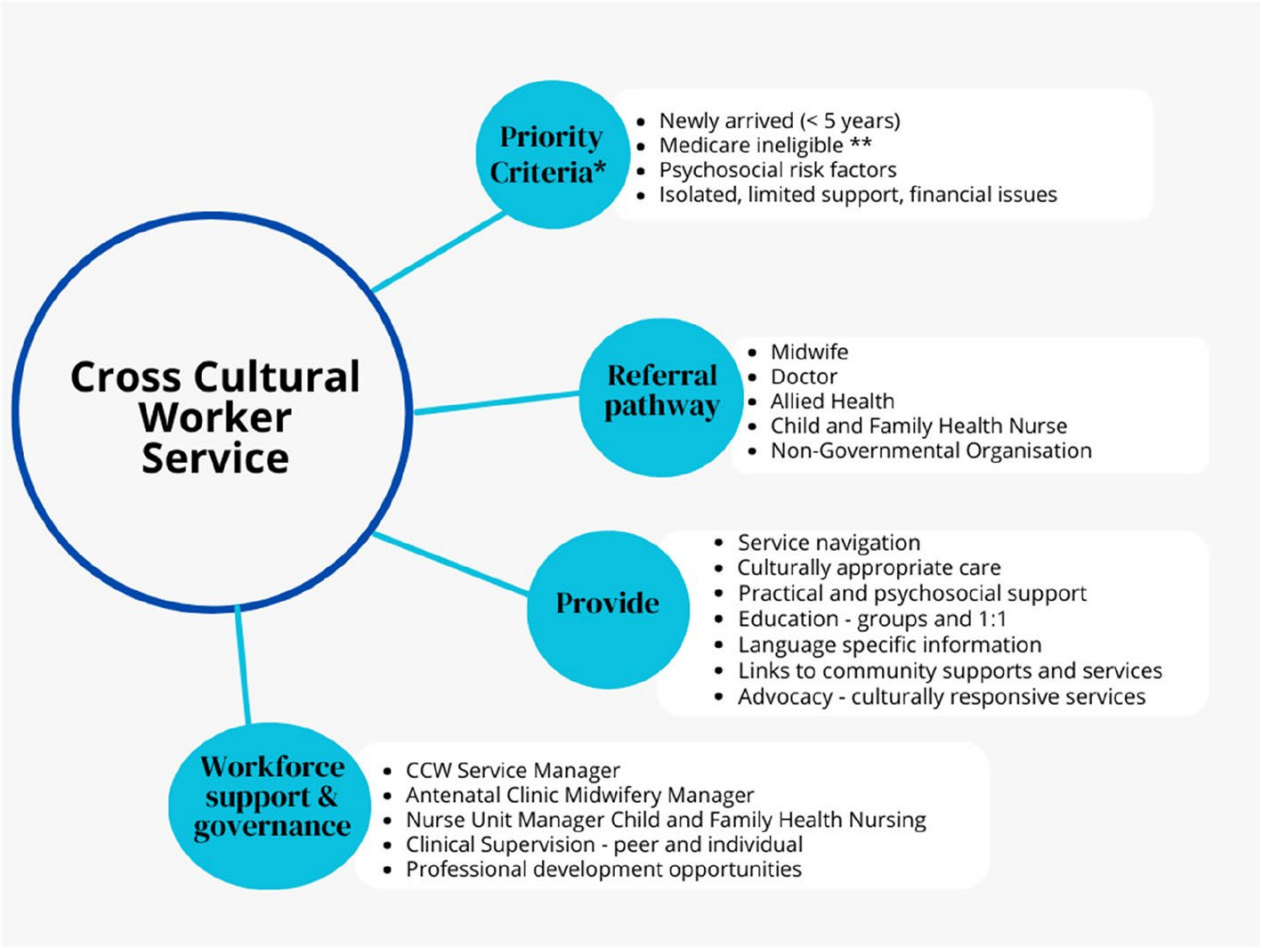
Cross cultural worker model of care. * All women and families from migrant and refugee backgrounds can access the CCW Service, however priority is given to listed criteria. ** Medicare is Australia’s publicly-funded universal health insurance scheme. It guarantees all Australians (and some overseas visitors) access to a wide range of health and hospital services at low or no cost [53]

The CCWs focus on supporting women and families from migrant and refugee backgrounds who are socially isolated, financially disadvantaged, have limited support and/or psychosocial risk factors. Priority, is given to families who have resided in Australia for less than five years and/or Medicare ineligible, however absence of these factors is not a reason for exclusion from the Service. The model of care and inclusion criteria for the CCW Service are detailed in Fig. 1.

CCWs are part of the multidisciplinary team with midwives, child and family health nurses (CFHN), doctors, allied health and other service providers. They a) support women and families to navigate maternity, CFH, and community-based services, b) enable early access and ongoing service engagement across the continuum of pregnancy and the transition to CFH services, c) provide culturally appropriate support to women and their families, defined as care that takes cultural preferences into account [54], and are aligned with World Health Organization’s (WHO) standards for improving quality of maternal and newborn care [55, 56]. CCWs act as cultural brokers, peer support workers, or mediators between women/families and service providers, to provide practical and psychosocial support, language specific information (when available), education of women and families and service providers, and d) client advocacy and collaboration with health services, local communities and agencies to build capacity to provide culturally responsive services. The CCWs do not provide legal or migrant rights advice, rather inform clients of appropriate support services. The CCWs do not replace accredited interpreters.

The CCWs are co-located in hospitals and community-based clinics. Referrals are received from midwives, doctors, CFHN, allied health and non-governmental organisations (NGO). The CCWs liaise and collaborate with existing service providers to best respond to the client’s individual needs. All client contact occurs during office hours. The number, frequency and type of interaction (including face-to-face, telehealth, telephone, text messaging, and email contact) varies depending on the preference and needs of each woman and family.

### Study recruitment

Women utilising the CCW Service during pregnancy were invited to participate. Recruitment commenced in February 2019 and continued until July 2021. The CCWs discussed the study during their usual interactions. All participants were provided with a written consent form, including assurance of the voluntary, anonymous and confidential nature of the study, and were aware that the CCWs would not see their surveys. The consent form was available in Bangla, Bahasa, and Nepali. For women who did not speak English, the CCW worked with an interpreter to explain the research, as is standard CCW Service practice.

### COVID-19 pandemic impact

Between March and August 2020, COVID-19 public health restrictions impacted on the CCW Service provision of care, and recruitment to the study. Although the CCW Service maintained client contact through telephone, texting, and telehealth, study recruitment ceased from March to August 2020. Recruitment resumed September 2020 and continued through to June 2021.

### Data collection

Participants were invited to complete an anonymous, unlinked survey at three timepoints, third trimester of pregnancy, 6 and 12 months postpartum. Partners of the women were invited to complete the survey when their baby was 6 months old.

The surveys were developed by the research team based on a literature search of perinatal health service evaluation by service users, local maternity and CFH services consumer evaluations, as well as clinical and research experience. The survey questions were reviewed and piloted by the three CCWs, members of the CCW Service Working Group, and three clinical colleagues, with minor modifications made following feedback. Service users were not involved in the design or pilot of the survey (See Additional file 1).

The survey collected demographic information; women and their partners’ country of birth, period of residence in Australia, main language spoken at home, and highest level of education. Multiple choice questions and free-text options related to pregnancy included, parity, gestation at first hospital antenatal clinic visit, antenatal model of care, gestation at first CCW meeting, number of CCW interactions, and usefulness of the Service.

Participants rated overall satisfaction with the CCW Service, perception of CCW Service usefulness, if the CCW Service met their needs regarding provision of information on a range of topics, whether the CCW clearly communicated this information, if the CCW Service was sensitive to their cultural needs, and recommendation of the CCW Service to family and friends. Two separate questions asked participants thoughts on becoming a mother or parent before and after meeting the CCW. Free text options requested detail of what they liked least and most about the CCW Service, and suggestions for improvement.

The estimated survey completion time was within 15 min. The survey was available in English, Bangla, Indonesian Bahasa and Nepali depending on participant preference. Surveys were distributed by the lead researcher (HJR) according to participant preference, either via email invitation with an anonymous/no identifying details generic SurveyMonkey^©^ link, hard copy (antenatal clinics only), or postal. Prior to survey distribution, HJR asked the CCW if there were any women who should not be invited to participate, and also accessed the electronic medical record of each participant to identify any adverse event or key considerations that would warrant non-participation in the survey, e.g., significant mental health concerns or perinatal loss. The CCW workers did not see the responses.

Distribution of the pregnancy survey commenced in March 2019. Three email reminders were sent at two-week intervals to balance the opportunity to participate against the burden of ongoing emails. Distribution of maternal and partner 6-month postpartum surveys commenced September 2019, and 12-month postpartum surveys commenced March 2020. Distribution of all surveys ceased January 2022.

### Data analysis

Surveys were analysed using IBM Statistical Package for Social Sciences (SPSS IBM Corporation, Armonk, NY), software version 26. Demographic data and responses to individual questions were analysed descriptively. The pregnancy surveys were analysed using Pearson’s Chi-Squared testing to examine difference in level of overall satisfaction and likelihood of recommending the service by factors thought likely to influence this and with sufficient numbers for subgroup analysis, namely 1) completion prior (≤ 10 March 2020) versus during the COVID-19 pandemic (≥ 11 March 2020), 2) years residence in Australia (≤ 2 years, 3–5 years, ≥ 6 years), 3) the three main countries of birth (Nepal, Bangladesh, Indonesia), 4) gestation at first CCW contact (≤ 19, ≥ 20 weeks), and 5) number of CCW interactions (1, 2, 3, ≥ 4). Subgroup analysis was agreed prior to commencement of data analysis, however adequate responses were required for this analysis to be meaningful. Initially planned analysis by parity therefore did not proceed as the vast majority of participants were primiparous.

Chi-squared testing was also used to examine difference in participant experience with the CCW Service across the three survey timepoints. Participant country of birth, years residence in Australia, gestation at first CCW interaction, number of CCW interactions, usefulness of the CCW Service, level of satisfaction, sensitivity to cultural needs, thoughts on becoming a mother or parent before and after meeting the CCW, and recommendation of Service were compared. A *p*-value of < 0.05 was considered statistically significant.

Free text survey responses as to what participants liked most and least, and recommendations for CCW Service improvement were analysed using numerical content analysis, which involves the researcher quantifying the presence of certain words, themes, or concepts within qualitative text data [57]. Categories with five or more participant responses are reported.

The study was approved by the South Eastern Sydney Local Health District Human Research Ethics Committee in October 2017, approval number: HREC 17/257 with site- specific approval for all sites.

## Results

**Fig. 2:**
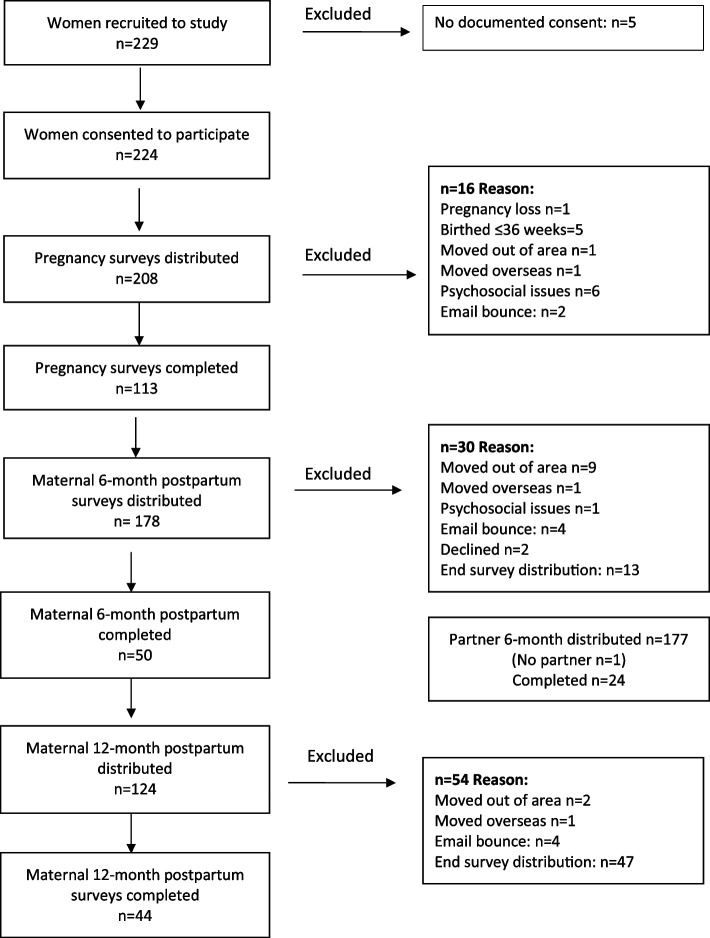
Flowchart of participants in the study

As shown in Fig. 2, a total of 229 women were recruited, and 45 women were excluded. In total, 231 survey responses were received; women during pregnancy (*n* = 113), at 6 months (*n* = 50) and 12 months (*n* = 44), and their partners (*n* = 24) when the baby was six months old. Due to research timeline and available resources, survey distribution ceased January 2022, which meant sixty women who completed the pregnancy survey did not complete six- and 12-month surveys. The survey response rate was 50% during pregnancy, 28% at 6 months, 36% at 12 months, and 20% for partners.

Thirty-nine participants (78%) completed both the pregnancy and 6-month postnatal survey, and 80% (*n* = 35) completed both the 12 month and pregnancy survey. Sixty participants (53%) completed the pregnancy survey prior to the COVID-19 pandemic in March 2020, and fifty-three women (47%) during the pandemic. The majority of the 6-month maternal surveys were completed during the pandemic (92%), as were all 12-month surveys.

### Women’s characteristics

Women reported 16 different countries of birth; for the pregnancy surveys, the highest frequencies were Nepal (47%), Indonesia (18%), and Bangladesh (15%). Most women (85%) had lived in Australia 5 years or less, spoke a language other than English at home (89%), were university educated (71%), and had not arrived in Australia as a refugee or asylum seeker (96%). On average, women reported that their partners had been in Australia 5 years or less (mean 4 years), however 25% had been in Australia 6 or more years and 4% were Australian-born (Table 1).

**Table 1:** Women’s demographics across all survey timepoints

Characteristic	Antenatal (*n* = 113) n (%)	6 months (*n* = 50) n (%)	12 months (*n* = 44) (n) %	*P* value
**Country of Birth**				**0.69**
Nepal	53 (47)	26 (52)	26 (59)	
Indonesia	20 (18)	2 (4)	5 (10)	
Bangladesh	17 (15)	5 (10)	6 (14)	
India	6 (5)	4 (8)	1 (2)	
Thailand	6 (5)	4 (0)	3 (7)	
China	2 (2)	0 (0)	0 (0)	
Mongolia	2 (2)	1 (2)	0 (0)	
Philippines	2 (2)	1 (2)	0 (0)	
Colombia	1 (1)	1 (2)	2 (5)	
Peru	1 (1)	1 (2)	1(2)	
Brazil	1 (1)	1 (2)	0 (0)	
Chile	1 (1)	1 (2)	0 (0)	
Spain	1 (1)	1 (2)	0 (0)	
Pakistan	0 (0)	1 (2)	0 (0)	
Kuwait	0 (0)	1 (2)	0 (0)	
Taiwan	0 (0)	0 (0)	0 (0)	
Australia	0 (0)	0 (0)	0 (0)	
**Years in Australia**	Mean 2.9 (SD 0.9)			**0.06**
≤ 2 years	26 (23)	12 (24)	7 (16)	
3–5 years	70 (62)	28 (56)	21 (47)	
≥ 6 years	17 (17)	10 (20)	16 (36)	
**Identify as refugee or asylum seeker**				**0.30**
Yes	0 (0)	0 (0)	0 (0)	
No	108 (96)	49 (98)	44 (100)	
Prefer not to say	5 (4)	1 (2)	0 (0)	
**Highest level of education**				**0.56**
Primary	0 (0)	1 (2)	0 (0)	
≤ Year 12	12 (11)	7 (14)	6 (14)	
Technical college	20 (18)	4 (8)	6 (14)	
University	80 (71)	38 (76)	32 (73)	
Other	1 (1)	0 (0)	0 (0)	
**Main language spoken at home**				**0.65**
English	13 (12)	8 (16)	7 (16)	
Other	100 (89)	42 (84)	37 (84)	
**Parity**				**0.39**
Primip	104 (92)	43 (86)	41 (93)	
Multip	9 (8)	7 (14)	3 (7)	
**Partner years in Australia**	Mean 4 (SD 1.02)			**0.27**
≤ 2 years	5 (5)	2 (4)	1 (2)	
3–5 years	30 (27)	11 (22)	6 (14)	
≥ 6 years	28 (25)	21 (42)	19 (43)	
Australian born	4 (4)	4 (8)	4 (9)	
No response	18 (16)	12 (24)	14 (32)	

Most women completed the pregnancy survey between 36 to 40 weeks gestation (69%), were expecting their first baby (92%) and attended their first antenatal hospital appointment at or before 19 weeks of pregnancy (73%). Just over a quarter of women (28%) had their first visit with the CCW between 15 and 19 weeks’ gestation. During pregnancy, the majority (55%) had four or more interactions with the CCW Service, and 36% three interactions.

### Women’s experience of the CCW Service

The vast majority of women during pregnancy found it useful to have the CCW provide information and resources (98%), this decreased somewhat to 84% at 6 months, and 86% at 12 months (*p* = 0.005). Women reported that the CCW Service supported them to understand information about pregnancy, birth and parenting. During pregnancy, women mostly reported that the CCW always provided high levels of culturally sensitive care (65%), and this was consistent in the 6 month and 12-month surveys. During pregnancy, CCWs provided women with the opportunity to ask questions at all or most interactions (80%). This continued after the baby was born albeit at slightly lower levels (70%) at 6 months, and (64%) at 12 months (*p* = 0.049).

Women reported high rates of satisfaction with the CCW service. (Table 2). Regarding factors potentially influencing satisfaction with and subsequent recommendation of the CCW service to family and friends, no statistically significant relationship was found between the level of satisfaction with the CCW Service and years residence in Australia (*p* = 0.69), or recommendation to family and friends (*p* = 0.15). Satisfaction and recommendation were also not related to country of birth, number of CCW interactions (*p* = 0.78), or gestation of first CCW interaction (*p* = 0.28). There was also no difference found in level of satisfaction with the CCW Service and number of CCW contacts (*p* = 0.95), or number of CCW contacts and engagement with the CCW Service pre-COVID (*p* = 0.83) or during the COVID-19 pandemic (*p* = 0.63). Women who were more satisfied with the CCW service were significantly more likely to recommend the service to family and friends across all three timepoints (*p* < 0.001).

**Table 2:** Women and Partners responses

Characteristic	Pregnancy (*n* = 113) n (%)	6 months (*n* = 50) n (%)	12 months (*n* = 44) n (%)	*p* value (Women)	Partner 6 months (*n* = 24) (n) %	*p* value (All participants)
**Was it useful to have the CCW talk to you and provide information/resources?**				**0.01**		**0.02**
No	2 (2)	2 (4)	1 (2)		0 (0)	
Yes	111 (98)	42 (84)	38 (86)		21 (88)	
Not sure/cannot remember	0 (0)	6 (12)	5 (11)		3 (12) N/A Did not talk to CCW	
**Supported understanding information to prepare for pregnancy, birth, or parenting**				**0.96**		**0.71**
Yes	113 (100)	48 (96)	42 (95)		22 (95)	
No	0 (0)	1 (2)	2 (5)		2 (5)	
N/A	0 (0)	1 (2)	0 (0)		0 (0)	
**Could you ask the CCW any questions you had**				**0.05**		** < 0.001**
Yes, a few times	20 (18)	13 (26)	10 (23)		5 (21)	
Yes, most of the time	34 (30)	14 (28)	15 (34)		6 (25)	
Yes, all of the time	56 (50)	21 (42)	13 (30)		11 (46)	
Not sure/don't know	1 (1)	1 (2)	4 (9)		0 (0)	
No, never	0 (0)	1 (2)	2 (5)		2 (8) Did not talk to CCW	
Missing	2 (2)	0 (0)	0 (0)		0 (0)	
**Sensitive to your cultural needs and those of your family**				**0.36**		**0.40**
Not sure/cannot remember	11 (10)	4 (8)	6 (14)		5 (21)	
No	3 (3)	6 (12)	3 (7)		2 (8)	
Yes, sometimes	24 (21)	8 (16)	8 (18)		3 (13)	
Yes, always	73 (65)	32 (64)	27 (61)		14 (58)	
Missing	2 (2)	0 (0)	0 (0)		0 (0)	
Not sure/cannot remember	11 (10)	4 (8)	6 (14)		5 (21)	
**Level of satisfaction**				**0.29**		**0.42**
Very dissatisfied	3 (3)	2 (4)	0 (0)		0 (0)	
Dissatisfied	1 (1)	0 (0)	1 (2)		0 (0)	
Neither satisfied/dissatisfied	3 (3)	4 (8)	0 (0)		1 (4)	
Satisfied	43 (38)	17 (34)	19 (43)		7 (29)	
Very satisfied	63 (56)	27 (54)	23 (52)		15 (63)	
Not sure/don’t know	0 (0)	0 (0)	1 (2)		1 (4)	
**Recommend CCW Service to friends and family**				**0.15**		**0.04**
Definitely won’t	0 (0)	0 (0)	1 (2)		1 (4)	
Maybe	3 (3)	4 (8)	0 (0)		0 (0)	
Probably will	16 (14)	9 (18)	8 (18)		2 (8)	
Definitely will	94 (83)	37 (74)	35 (80)		20 (83)	
Not sure/don’t know	0 (0)	0 (0)	0 (0)		1 (4)	

Women reported that meeting the CCWs made them feel less nervous about becoming a mother, 33% before meeting to 3% afterwards (*p* < 0.001). Meeting the CCW also made women feel more excited about becoming a mother, 44% before meeting to 75% afterwards (*p* < 0.001). These women’s perceptions on becoming a mother before and after meeting the CCW were retained in the postnatal period (See Additional file 2 Table S2 for detail).

Women felt that the CCW service met their needs in the provision of information during pregnancy on fourteen topics. Thirteen out of fourteen topics had a median score of 5 “a lot” (IQR 3–5, mean 4.2–4.8, SD 0.9–1.2). The topic of financial entitlements, rated slightly lower with a median score 4 (IQR 3–5, mean 3.9, IQR 1.3). Most women felt the information was clearly communicated by the CCW (*n* = 96, 85%, median 5) (See Additional file 2 Table S3 for detail).

Additionally, the 6- and 12-month surveys rated the effectiveness of the CCW role meeting women’s needs in the provision of information on eleven topics related to parenting, including available services and supports, feeding and caring for baby. In the 6-and 12-month surveys, seven topics had median scores of 5 being “a lot” (IQR 3–5). Similar to during pregnancy, women wanted more information on financial entitlements (median 4 at 6 months, and 3 at 12 months 3–4, IQR 2–5). For 6- and 12-month surveys women wanted more information on emotional support and support networks (median 4, IQR 3–5). At 6 months postpartum, women also wanted more information on caring for their baby (median 4, IQR 3.8–5), and community supports in their local area (median 4, IQR 3–5). At 12 months postpartum, women wanted more information on feeding their baby (median 4, IQR 3–5), and becoming a parent (median 4.5, IQR 3–5). Most women felt information was clearly communicated by the CCW (median 5, IQR 4–5) (See Additional file 2 Table S4 and S5 for detail of topics and ranking).

### Partner surveys

A total of 24 partners completed the 6-month postpartum survey from eight countries of birth; the majority being born in Nepal (50%), Bangladesh and Australia (12%). Partners had resided in Australia for an average of 5.9 years (SD 0.9), 74% spoke a language other than English at home, and the majority were university educated (62%). Many (38%) did not identify as belonging to a cultural or ethnic group, and none identified as being a refugee or asylum seeker. For most partners (92%), it was their first baby (92%). Most partners met the CCW for the first-time during pregnancy between 15- and 24-weeks’ gestation (66%), on three or more occasions (66%), while 21% had not met the CCW (See Additional file 2 Table S1 for detail).

Most partners found it useful to have the CCW provide information and resources (88%) and the CCW supported them to understand information to prepare for parenting (96%). All partners who met the CCW felt welcome and involved in conversations, and that they could ask any questions they had on all or most CCW interactions (71%). Partners reported that the CCW provided culturally sensitive care on most occasions (71%). Almost all (92%) partners were satisfied or very satisfied with the CCW Service and would recommend it to family and friends. Similar to the women’s surveys, partners who were more satisfied were more likely to recommend to family and friends (*p* < 0.001), and the number of CCW interactions did not impact on this. Partners also reported that meeting the CCW made them feel more excited about becoming a parent, although this did not reach statistical significance (59% before meeting to 75% afterwards, *p* = 0.45) (Table 2).

The effectiveness of the CCW Service meeting partners’ needs in the provision of information, twelve of fourteen topics had a median score of 5 (“a lot”), including health services and facilities, child development, feeding baby, emotional support and networks, and financial entitlements/support. Topics with slightly lower scores were financial entitlements (median 3.5), and emotional support and networks (median 4.5). Most partners felt information was clearly communicated by the CCW (median 4). (See Additional file 2 table S6 for detail of topics and ranking).

### Free text responses: Liked most, least, and recommendations for CCW Service improvement

A total of 192 responses described the CCW Service aspects they liked most as information and knowledge sharing (*n* = 67), the opportunity to talk, share issues and ask questions (*n* = 32), the CCWs supportive, helpful nature (*n* = 61), and friendliness (*n* = 26). Participants also liked that the CCW Service clearly communicated information that was easy to understand (*n* = 11), and shared culture (*n* = 8). Women and their partners expressed this by stating:

*They are excellent, friendly, helpful. The information and resources were good. It helps us to prepare physically, mentally* (Maternal pregnancy survey).

*Before I didn't have knowledge regarding pregnancy, birth, and parenting but now I have knowledge regarding pregnancy, birth, and parenting which makes me confident, and to cope with all these changes smoothly.* (Maternal pregnancy survey)

*Helped make my wife feel very comfortable about accessing available resources in what was otherwise a foreign environment having only arrived in Australia last year and where English is not her first language* (Partner 6-month survey).

Aspects liked least about the CCW Service received 20 responses. Participants wanted more education and information (*n* = 14) specifically related to financial entitlements, postnatal depression, and support services. A total of 39 responses were received in relation to suggestions for CCW Service improvement, including, increase provision of information (*n* = 17), increase the CCW workforce (*n* = 8), and involvement of partners (*n* = 5). Women and their partners expressed this by stating:

*Need more sessions regarding postnatal depression for both father and mother and basically more about accessing Centrelink and social support for the families* (Maternal pregnancy survey).

*To improve Cross Cultural Worker Service, I think there should be more volunteers and more lessons. Like how to be Dad class, how they can help their partners during and after pregnancy* (Mother 6-month survey).

## Discussion

This study provides insight into the perspectives of women from migrant and refugee backgrounds and their partners accessing the CCW Service during pregnancy through to 12-months postpartum.

Women reported high levels of satisfaction and would recommend the CCW Service to family and friends across all three survey timepoints. The high level of satisfaction and CCW Service recommendation was not substantively affected by country of birth, number of CCW interactions, or engagement with CCW Service pre or during the COVID-19 pandemic. The CCW Service was perceived by women and their partners to be effective in supporting access to information and education, and their understanding of information which improved their pregnancy and parenting experience. Importantly, women’s thoughts on becoming a mother or parent were significantly more positive after meeting the CCW than before. Suggestions for Service improvement from a minority of participants included greater involvement of partners in care, increased information on financial, postnatal depression, and support services, and increasing the CCW workforce.

### Overview of findings in relation to prior studies

Women from migrant and refugee backgrounds settling in HIC have reported dissatisfaction with their maternity care [27, 58, 59]. Women and their partners in our study reported high levels of satisfaction, providing new evidence of the benefits of Cross Cultural/bicultural/bilingual workers supporting women and families from migrant and refugee backgrounds. A recent systematic review of models of care in this field [38], identified nine studies that evaluated the impact of inclusion of bicultural/bilingual/multicultural/peer workers in perinatal service provision [39–44, 46, 47, 49]. The CCW Service engaged similar approaches reported in the literature, including information exchange [39, 42, 44, 46, 49], continuity of care [40, 42, 44], provision of a social model of care [39, 42, 44, 47], which enhanced communication between women and service providers [39, 42, 44, 45], and cultural sensitivity of service providers [39–44, 46, 49]. Of the nine studies in the review, five sought service users’ perspectives to evaluate the effectiveness of the model of care [39, 40, 42, 44, 49]. Women’s perceptions of a community-based group model of pregnancy care for refugee women described the practical and emotional support provided by a bicultural worker within the multidisciplinary team, ensured cultural safety [39]. Similar to our findings regarding the CCW Service, women described that the provision of information by bicultural/bilingual workers was highly valued, increased their confidence, and enabled preparation for birth [39, 40], and parenting [39, 42, 44, 49].

The benefits of bicultural workers are also described by Lutchenbacher et al. [43] in a randomised controlled trial of bicultural peer mentors with a similar scope of practice to the CCWs in provision of health education, psychosocial support, and referral to community supports. This study reported the intervention group had a higher retention to breastfeed, fewer depressive symptoms, less parenting stress, better safe sleep practices, and infant stimulation in the home [43]. Women also reported high levels of satisfaction with the intervention [43]. Another study using bicultural/bilingual workers to provide antenatal education and navigation support for Latino women reported increased understanding of the importance of antenatal care, signs of preterm birth, breastfeeding benefits, safe sleeping, shaken baby syndrome and normal mood changes [60].

In a recent study of service providers perceptions of the CCW Service, the CCWs lived experience of the migration and settlement journey, shared language, and cultural understanding was seen as integral to the provision of continuity of carer, culturally appropriate care that supported service engagement, and confidence to access services independently [61]. Similarly, the support of multicultural doulas in Norway was reported to strengthen maternity care for migrant women by providing information, continuity, and a cultural bridge between migrant women and maternity care [62]. Links with bicultural peer workers, particularly from the same cultural group, already more accustomed to the host country, and who have often endured identical or similar situations can play a significant role, as sharing experiences mitigates isolation, discouragement, and anxiety [63].

Previous studies have recommended the need to explore migrant partners experiences [5, 51, 64, 65]. Of the few previous studies [66, 67], although partners (*n* = 27) were positive about their interactions with health services, it was evident services were not always responsive to issues related to settlement in a new country, and stresses associated with the transition to parenting. Similar to our study, services did not always enable partners’ involvement, or the opportunity to access information and ask questions. Limited CCW interaction with partners, particularly in the context of the COVID-19 pandemic, may have resulted in partners not feeling included, as reflected in a lower partner response rate. The level of participation may have been higher for partners who were more engaged with the CCW Service, hence the positive partner survey responses in our study do need to be interpreted with caution. However, under pre-pandemic conditions, research suggests that partners often feel excluded from maternity and early parenting services, [68] and changes to care during the pandemic may have exacerbated exclusion [69]. The provision of targeted culturally responsive, family inclusive services for families from migrant and refugee backgrounds are integral to paternal and maternal health literacy and mental health [66, 67].

In the current study, women and their partners main suggestion to improve the CCW Service was to increase the CCW workforce. This aligns to those of service providers, which highlighted that in order to meet client demand, and maintain a balance between maternity and CFH services, an increase in workforce was essential [61]. The complexity of navigating health services, particularly the transition between maternity and CFH services, are key barriers to access for women and families from migrant and refugee backgrounds [22]. Bicultural/bilingual workers have demonstrated their positive contribution to improving access to maternity and CFH services through navigation support [39, 43, 44, 60, 61, 70, 71], fostering relationships between service providers and service users to improve the cultural responsiveness of services [43, 60, 61, 72]. Their support and education can increase health literacy, reinforce the importance of maternal and CFH health, and child developmental assessments, in order to optimise health outcomes [22, 73]. Although the ability of bicultural/bilingual workers to improve service access and the care experience are well documented, evidence to demonstrate the impact on improvements in maternal and infant health outcomes is required [38, 74]. Importantly, memories associated with pregnancy, birth, and early parenting are often powerful and long lasting [75], these experiences can influence how women and families perceive and engage with health services in the future [22, 76].

### Strengths and limitations of the study

One strength of this study is the perspectives of women in pregnancy as well as 6 and 12 months postpartum. Although fewer responses were received in the postpartum period, over three quarters who completed the pregnancy survey completed at least one postnatal survey (self-report). Another strength of the study is the partner survey. Partner experiences are particularly important as many people from migrant and refugee backgrounds come from collectivist communities in which decisions are shared and involve family and community. Although only twenty-four responses were received, their perspective helps in understanding the parenting needs of fathers from migrant backgrounds, to inform the CCW service and future care.

A further strength of the study was that the surveys were translated to increase participation. Additionally, by conducting an anonymous online survey, the identity of the participants was protected, which aimed to facilitate reporting honest perspectives. The study is limited by the relatively small sample in relation to the available study population (50% of pregnancy cohort however lower for postnatal surveys). Factors influencing response rate included that due to research timeline (in the setting of a continuing/ongoing Service) the distribution of six- and 12-month surveys ended in January 2022, excluding 60 women from postnatal survey completion. Additionally, the COVID-19 pandemic disrupted recruitment to the study, and may have also reduced participation, due to the additional stressors experienced by migrant and refugee communities, including increased isolation due to enforced lockdowns and travel restrictions, unemployment, and financial hardship [77–80].

The findings predominantly capture the views of women born in South Asia, and who did not identify as being a refugee or asylum seeker. Although this aligns to the communities accessing the study hospitals, it may limit generalisability and comparability of findings to other migrant and refugee populations. In addition, most participants (71–76%) were university-educated, higher than the overall population of women aged 25–44 years in Australia (50%) [81], however comparable to recent arrivals to Australia, of whom 79% had a Bachelor Degree or higher [82]. We acknowledge that women with lower educational level, lower socioeconomic background, and/or less positive experience of care, may have been less likely to complete the survey.

Another potential limitation of the study was the involvement of the CCW in recruitment of participants. Although the survey was anonymous, and study information and recruitment conversations reinforced this, and that survey findings would have no impact on the CCW employment, or participant current and future care, some participants may have been hesitant to voice dissatisfaction or concerns about their care and the CCW Service and chosen not to participate. The lead researcher (HJR) perceived that the trusting relationship, and shared migration journey, between CCWs and potential participants would ensure accessible and culturally appropriate information about the study was given, providing a sense of trust and safety in participation. Studies have also reported that data collected may be enhanced through involving trusted workers in the process [39, 83]. However, we are cognizant this approach has the potential for respondent bias and favorable feedback. Furthermore, as surveys were anonymous and unlinked, it was not possible to match responses, or clarify any potential data ambiguity.

A further potential limitation of the study was the survey instrument used. Although the survey was piloted by three CCWs, members of the CCW Service Working Group, and three clinical colleagues, measures were not formally validated. A culturally validated Migrant Friendly Maternity Care Questionnaire (MFMQ) [84] has been developed to measure migrant-sensitive care across a range of maternity care settings and countries. However, we decided not to use the MFMQ for this study as it was developed for administration to women four months post birth via interview, rather than a written survey at various timepoints during pregnancy and postpartum to allow women to reflect progressively on their care experiences, for which to our knowledge there is no validated measure available. Additionally, at 112 items the MFMQ length was felt to be prohibitively long for our participants, given that client experience and satisfaction with care requires brief measures for use generally and with specific populations to inform practice [66, 67]. However, we did include similar questions to the MFMQ related to migration status, access to care, perceptions of care, caregiver responsiveness, and information exchange, as well as bespoke questions specific to our non-clinical model of care and CCW scope of practice.

### Implications for practice

The findings from this study of the CCW Service highlights how health services can provide an individualised approach that responds to the complex perinatal needs of women and families from migrant and refugee backgrounds. Our findings support Australian antenatal care guidelines [85], and World Health Organization guidelines [10] that recommend models of care that involve bicultural/bilingual workers ensure to ensure culturally responsive service provision. The current and future study findings have the potential to support scalability and sustainability in high income countries with adaptations based on local settings and communities. Migrant women and families are not a homogenous group, workers with shared language and culture may be required to support local priority populations with significant adverse outcomes. However, the relevance for a particular cultural or language should be carefully considered to avoid “othering” of women into risk groups [86].

Service provision needs to draw on consultations with migrant and refugee communities to co-design services, and to obtain a greater understanding of socioeconomic and cultural factors, health beliefs and practices that impact on service access [38]. Furthermore, integral to service provision, is consideration of the key elements of acceptable and accessible models, including, culturally responsive continuity of care, effective communication, psychosocial and practical support, support to navigate systems, in-language information and access to interpreters, trauma-informed care, affordable services, a social model of care, co-design with local communities, flexible and accessible services [38].

Sustainability of the CCW Service and similar models of care, relies on maintaining the balance of client workload, advocacy, professional development, and governance. There is also the demand for cultural expertise, co-design, community engagement and collaborative projects with key stakeholders, which is limited by part-time hours. Consequently, adequate staffing, clinical supervision, and effective management are imperative to support and retain the bicultural/bilingual workforce [61]. To date, our research findings have supported embedding the model of care in the study sites by changing the CCW positions from contract to permanent, and an increase CCW hours has enabled the Service to improve support postnatally. We recommend further research strengthen understanding of targeted models of care include the perceptions of partners, who frequently feel excluded from maternity and early parenting services.

## Conclusion

The CCW Service was associated with positive experience and high rates of satisfaction across all timepoints. Women and their partners reported the Service increased their understanding of pregnancy, birth and parenting, and that they would recommend the CCW Service to family and friends. Thoughts on becoming a mother or parent were significantly more positive after meeting the CCW than before for both women and partners. Recommendations to improve the Service were to increase the provision of information, involvement of partners in care, and the CCW workforce. This tailored service has the potential to inform the implementation of similar models of care that improve accessibility, the perinatal experience, and respond to the unique needs of women and families from migrant and refugee backgrounds.

### Supplementary Information

**Additional file 1.**

**Additional file 2.**

**Additional file 3.**

## Data Availability

Datasets are available from the corresponding author on reasonable request.

## Abbreviations

CCWs: Cross Cultural Workers
The CCW Service: The Cross Cultural Worker Service
CFHN: Child and Family Health Nurse
NGO: Non-Governmental Organisation
HJR: Helen J Rogers
AH: Amanda Henry
CH: Caroline Homer
