# Supplementary material for: Perspectives of women and partners from migrant and refugee backgrounds accessing the Cross Cultural Worker Service in maternity and early childhood services—a survey study
Source: BMC Health Serv Res. 2023 Nov 10;23:1233. doi: 10.1186/s12913-023-10194-3 (PMC10636916; doi:10.1186/s12913-023-10194-3)
Supplement: Supplementary file 1 — Additional file 1. [file 12913_2023_10194_MOESM1_ESM.pdf]

Additional file 1:

Perspectives of women and partners from migrant and refugee backgrounds accessing the Cross Cultural Worker Service in maternity and early childhood services - a survey study

Authors' list

Helen J. Rogers PhD Candidate, RM, RN, MPH [1,2]

Professor Caroline SE Homer AO RM PhD [3,4]

Associate Professor Amanda Henry PhD MPH FRANZCOG BMed BMedSci DDU (O&G) [2,5,6]

Author affiliations

[1] Child, Youth & Family Services, South Eastern Sydney Local Health District, Sydney, NSW, 2010, Australia

[2] Discipline of Women's Health, School of Clinical Medicine, University of NSW (UNSW), Sydney, NSW, 2000, Australia

[3] Maternal and Child Health, Burnet Institute, Melbourne, Vic, 3004, Australia

[4] Centre for Midwifery and Child and Family Health, Faculty of Health, University of Technology Sydney, NSW, 2007, Australia

[5] Department of Women's and Children's Health, St George Hospital, Sydney, NSW, 2217, Australia

[6] Australia Global Women's Health Program, The George Institute for Global Health, Sydney, NSW, 2042, Australia

Additional file 1:

Survey for women during pregnancy

Survey for women 6 months postpartum

Survey for women 12 months postpartum

Survey for partners baby 6 months old

English - Cross Cultural Workers in Maternity and Child & Family Health Services (Pregnancy)

Pregnancy Evaluation

Your experience of maternity and child and family health care is very important to us.

You have been invited to complete this survey because you have used the Cross Cultural Workers in Maternity and Child & Family Health Services. We are reviewing the Service and would like you to give us some feedback about your experience using the Service. Your experience is important as it helps us understand the quality of care you received and allows us to see where we need to improve.

Taking part in the survey is voluntary. We respect your decision to either complete or not complete the survey. If you decide not to participate, it will not affect the care you receive now or in the future. Whatever your decision, it will not affect your relationship with the staff caring for you.

Any information obtained will remain private and confidential. The survey will take about 10-15 minutes to complete. It will ask questions about you and your experience using the Cross Cultural Worker Service.

Thank you in advance for completing the survey, we greatly appreciate your feedback to help us improve service provision. We will also seek your feedback of the Cross Cultural Worker Service again when your baby is 6 and 12 months old and greatly appreciate any feedback you provide.

Researcher:Helen Rogers - Early Parenting Program Coordinator
Child, Youth & Family Services
South Eastern Sydney Local Health District Health
Email: Helen.Rogers@health.nsw.gov.au

* 1. What is the name of the country where you were born?

____

* 2. Did you arrive in Australia as a refugee or asylum seeker?

- ◯ Yes
- ◯ No
- ◯ Prefer not to answer

* 3. How long (years/months) have you lived in Australia?

____

4. Do you belong/identify with a cultural or ethnic group/s? If so, which one/s?

* 5. What is the name of the country where your partner was born?

Name of the country?

If born overseas, how long have they lived in Australia?

* 6. Which language do you mainly speak at home?

- English
- Other (please specify)

* 7. What is the highest level of education you have **completed?**

- Primary school
- Secondary to Year 12 or equivalent
- Technical college e.g. TAFE or equivalent
- University
- None of the above
- Other (please specify)

* 8. How many weeks pregnant are you today?

* 9. Is this your first baby?

- Yes
- No

10. If not your first baby, how many children do you have?

- ◯ 1
- ◯ 2
- ◯ 3
- ◯ 4 or more

11. What are the age(s) of your children (if applicable)?

* 12. How many weeks pregnant were you when you attended the hospital antenatal clinic in Australia for the **first time**?

- ◯ Less than 14 weeks pregnant
- ◯ 15 - 19 weeks pregnant
- ◯ 20 - 24 weeks pregnant
- ◯ 25 - 28 weeks pregnant
- ◯ 29 - 32 weeks pregnant
- ◯ 33 or more weeks pregnant
- ◯ Not sure/cannot remember

* 13. Have you received antenatal (pregnancy) care, from **any** of the following? Please select all that apply.

- ☐ Hospital midwife/midwives' clinic
- ☐ Midwifery Group Practice
- ☐ Antenatal Clinic/High risk clinic hospital doctors
- ☐ Private obstetrician
- ☐ GP/Family Doctor
- ☐ Shared Care
- ☐ Other, who

* 14. If you have received antenatal (pregnancy) care, what has been your **main** model of antenatal care? Please select **one** option.

- ◯ Hospital midwife/midwives' clinic
- ◯ Midwifery Group Practice
- ◯ Antenatal Clinic/High risk clinic hospital doctors
- ◯ Private obstetrician
- ◯ GP/Family Doctor
- ◯ Shared Care
- ◯ Other, who

* 15. How many weeks pregnant were you when you met the **Cross Cultural Worker** for the **first time**?

- ◯ Less than 14 weeks pregnant
- ◯ 15 - 19 weeks pregnant
- ◯ 20 - 24 weeks pregnant
- ◯ 25 - 28 weeks pregnant
- ◯ 29 - 32 weeks pregnant
- ◯ 33 or more weeks pregnant
- ◯ Not sure/cannot remember

* 16. How many times did you talk with the Cross Cultural Worker during your pregnancy so far (includes telephone, face to face, groups)?

- ◯ 1
- ◯ 2
- ◯ 3
- ◯ 4 or more
- ◯ Not sure/cannot remember

17. How prepared did you feel for your labour and birth?

1=Not prepared at all. 3=Neutral. 5=Well prepared. N/A=Not applicable.

1. Not prepared at all	2	3. Neutral	4	5. Well prepared	N/A
◯	◯	◯	◯	◯	◯

* 18. Was it useful to have the Cross Cultural Worker talk to you and provide information and resources?

- ◯ Yes
- ◯ No

If you have any comments please provide

* 19. Do you think the Cross Cultural Worker Service has supported you to understand information to prepare for pregnancy, birth and parenting?

- ◯ Yes
- ◯ No
- ◯ Not applicable

If you have any comments please provide

20. Did you feel you could ask the Cross Cultural Worker any questions you had?

- ◯ No, never
- ◯ Yes, a few times
- ◯ Yes, most of the time
- ◯ Yes, all of the time
- ◯ Not sure/don't know

If you have any comments please provide

* 21. Did the Cross Cultural Worker Service meet your needs providing information on the following?

1=Not at all. 3=Neutral. 5=A lot. N/A=Not applicable

	1. Not at all	2	3. Neutral	4.	5. A lot	N/A
Health services and facilities	○	○	○	○	○	○
Child and family health services	○	○	○	○	○	○
Antenatal and parenting education	○	○	○	○	○	○
Where to find more information and support	○	○	○	○	○	○
How your partner could provide support	○	○	○	○	○	○
Child development	○	○	○	○	○	○
Entitlements to financial support, e.g. Centrelink, etc	○	○	○	○	○	○
Emotional changes in pregnancy and after the birth	○	○	○	○	○	○
Physical changes in pregnancy and after the birth	○	○	○	○	○	○
Feeding your baby	○	○	○	○	○	○
Caring for your baby	○	○	○	○	○	○
Becoming a parent	○	○	○	○	○	○
Community supports, playgroups, services in your local area	○	○	○	○	○	○
Emotional support and support networks	○	○	○	○	○	○

* 22. In relation to question 21 overall, did you feel the Cross-Cultural Worker **communicated clearly** about the information she provided to you?

1=Not at all. 3=Neutral. 5=Very well. N/A=Not applicable

1. Not at all		3. Neutral	4	5. Very well	N/A
○	○	○	○	○	○

If you have any comments please provide:

* 23. How would you describe your experience with babies less than 6 weeks old?

- Lots of experience
- Some experience
- Very little experience
- No experience

* 24. Which one word would best describe your thoughts about becoming a Mother **before** you met the Cross Cultural Worker?

- Very nervous
- Nervous
- OK/Neutral
- Excited
- Very excited
- Not sure/don't know

* 25. Which one word would best describe your thoughts about becoming a Mother **after** you met the Cross Cultural Worker?

- Very nervous
- Nervous
- OK/Neutral
- Excited
- Very excited
- Not sure/don't know

* 26. Do you think the Cross Cultural Worker Service had any impact on your pregnancy experience?

- Not at all
- A little
- A lot
- Not sure/don't know

If so how, please provide any detail:

27. Was the Cross Cultural Worker sensitive to your cultural needs and those of your family?

- Yes, always
- Yes, sometimes
- No
- Not sure/cannot remember

If you have any comments please provide:

* 28. How would you rate your level of satisfaction with the Cross Cultural Worker Service?

- Neither satisfied nor dissatisfied
- Satisfied
- Very satisfied
- Dissatisfied
- Very dissatisfied
- Not sure/don't know

If you have any comments please provide:

29. What did you like **most** about the Cross Cultural Worker Service?

30. What did you like **least** about Cross Cultural Worker Service?

31. If we were to improve the Cross Cultural Worker Service, what do you think we should change?

* 32. Would you recommend this service to your friends and family?

- ◯ Definitely won't
- ◯ Probably won't
- ◯ Maybe
- ◯ Probably will
- ◯ Definitely will
- ◯ Not sure/don’t know

Please provide any additional comments you would like to make:

Please follow the link below to provide name and telephone number if you would like to volunteer to be selected to also participate in a telephone conversation related to the Cross Cultural Workers in Maternity and Child & Family Health Service. The discussion would be approximately 30-45 minutes and give us more detail to help us improve our services. As stated earlier, your privacy will be protected.
Link to volunteer to participate in telephone conversation: *SESLHD-CrossCulturalWorkers-Maternity&ChildAndFamilyHealth@health.nsw.gov.au*

Thank you for your time and participation!

English - Cross Cultural Workers in Maternity and Child & Family Health Services (6 months)

6 Months Postnatal Evaluation

Your experience of maternity and child and family health care is very important to us.

You have been invited to complete this survey because you have used the Cross Cultural Workers in Maternity and Child & Family Health Services. We are reviewing the Service and would like you to give us some feedback about your experience using the Service.

If you have already completed our survey in your pregnancy, Thank You! Please complete this follow up survey too as it asks about your experience of the Cross Cultural Worker Service since the birth of your baby. We will also seek your feedback again when your baby is 12 months old. Your feedback is very important as it helps us understand the quality of care you received and allows us to see where we need to improve.

Taking part in the survey is voluntary. We respect your decision to either complete or not complete the survey. If you decide not to participate, it will not affect the care and support you receive now or in the future. Whatever your decision, it will not affect your relationship with the staff caring for you.

Any information obtained will remain private and confidential. The survey will take about 10-15 minutes to complete.

Thank you in advance for completing the survey, we greatly appreciate your feedback to help us improve our Service.

Researcher:Helen Rogers - Early Parenting Program Coordinator
Child, Youth & Family Services
South Eastern Sydney Local Health District Health
Email: Helen.Rogers@health.nsw.gov.au

* 1. When did you first see the Cross Cultural Worker?

- ◯ During Pregnancy
- ◯ After the birth of my baby

* 2. If you first saw the Cross Cultural Worker during pregnancy, have you already completed the pregnancy survey? (it would have been given to you at about 36 weeks of pregnancy).

- ☐ Yes
- ☐ No

* 3. What is the name of the country where you were born?

[ ]

* 4. Did you arrive in Australia as a refugee or asylum seeker?

- ◯ Yes
- ◯ No
- ◯ Prefer not to answer

* 5. How long (years or months) have you lived in Australia?

6. Do you belong/identify with a cultural or ethnic group/s? If so, which one/s?

* 7. What is the name of the country where your partner was born?

Name of the country?

If born overseas, how long have they lived in Australia?

* 8. Which language do you mainly speak at home?

- ◯ English
- ◯ Other (please specify)

* 9. What is the highest level of education you have **completed?**

- ◯ Primary school
- ◯ Secondary to Year 12 or equivalent
- ◯ Technical college e.g. TAFE or equivalent
- ◯ University
- ◯ None of the above
- ◯ Other (please specify)

* 10. Is this your first baby?

- Yes
- No

11. If not your first baby, how many children do you have?

- 1
- 2
- 3
- 4 or more

12. What are the age(s) of your children (if applicable)?

* 13. How many weeks pregnant were you when you attended the hospital antenatal clinic in Australia for the **first time**?

- Less than 14 weeks pregnant
- 15 - 19 weeks pregnant
- 20 - 24 weeks pregnant
- 25 - 28 weeks pregnant
- 29 - 32 weeks pregnant
- 33 or more weeks pregnant
- I did not receive any antenatal care in Australia
- Not sure/cannot remember

* 14. How many weeks pregnant were you when you met the Cross Cultural Worker for the **first time**?

- Less than 14 weeks pregnant
- 15 - 19 weeks pregnant
- 20 - 24 weeks pregnant
- 25 - 28 weeks pregnant
- 29 - 32 weeks pregnant
- 33 or more weeks pregnant
- Baby less than 2 weeks old
- Baby between 2 weeks and 3 months old
- Baby older than 3 months
- Not sure/cannot remember

* 15. How old is your baby today?

* 16. How many weeks pregnant were you when you had your baby?

* 17. How many times did you talk with the Cross Cultural Worker **since your baby has been born** (includes telephone, face to face, groups)?

- ◯ 1
- ◯ 2
- ◯ 3
- ◯ 4 or more
- ◯ Not sure/cannot remember

* 18. Have you seen **any** of the following for postnatal care for you and your baby? Please select all that apply.

- ☐ Not receiving any postnatal care
- ☐ Child and Family Health Nurse
- ☐ GP/Family Doctor
- ☐ Other, who

19. **If you are receiving postnatal care**, who is providing **most** of the care for you and your baby? Please select only one.

- ◯ Child and Family Health Nurse
- ◯ GP/Family Doctor
- ◯ Other, who

* 20. How prepared did you feel for becoming a parent?

1=Not prepared at all. 3=Neutral. 5=Well prepared.

1	2	3	4	5
◯	◯	◯	◯	◯

* 21. Was it useful to have the Cross Cultural Worker talk to you and provide information in the previous 6 months?

◯ Yes

◯ No

◯ Not sure/cannot remember

If you have any comments please provide

* 22. Do you think the Cross Cultural Worker Service has supported you to understand information to prepare for parenting?

◯ Yes

◯ No

◯ Not applicable

If you have any comments please provide

* 23. Did you feel you could ask the Cross Cultural Worker any questions you had?

◯ No, never

◯ Yes, a few times

◯ Yes, most of the time

◯ Yes, all of the time

◯ Not sure/don't know

If you have any comments please provide

* 24. Did the Cross Cultural Worker Service meet your needs providing information on the following?

1=Not at all. 3=Neutral. 5=A lot. N/A=Not applicable

	1. Not at all	2	3. Neutral	4	5 A lot	N/A
Health services and facilities	◯	◯	◯	◯	◯	◯
Child and family health services	◯	◯	◯	◯	◯	◯
Where to find more information and support	◯	◯	◯	◯	◯	◯
How your partner could provide support	◯	◯	◯	◯	◯	◯
Child development	◯	◯	◯	◯	◯	◯
Entitlements to financial support, e.g. Centrelink, etc	◯	◯	◯	◯	◯	◯
Feeding your baby	◯	◯	◯	◯	◯	◯
Caring for your baby	◯	◯	◯	◯	◯	◯
Becoming a parent	◯	◯	◯	◯	◯	◯
Community supports, playgroups, services in your local area	◯	◯	◯	◯	◯	◯
Emotional support and support networks	◯	◯	◯	◯	◯	◯

* 25. In relation to question 24 overall, did you feel the Cross-Cultural Worker **communicated clearly** about the information she provided to you?

1=Not at all. 3=Neutral. 5=Very well. N/A=Not applicable

1	2	3	4	5	N/A
◯	◯	◯	◯	◯	◯

If you have any comments please provide:

* 26. Before you had your baby how would you describe your experience with babies **less than 6 months old**?

◯ Lots of experience

◯ Some experience

◯ Very little experience

◯ No experience

* 27. Which one word would best describe your thoughts about becoming a Mother **before** you met the Cross Cultural Worker?

- Very nervous
- Nervous
- OK/Neutral
- Excited
- Very excited
- I do not remember

* 28. Which one word would best describe your thoughts about becoming a Mother **after** you met the Cross Cultural Worker?

- Very nervous
- Nervous
- OK/Neutral
- Excited
- Very excited
- I do not remember

* 29. Do you think the Cross Cultural Worker Service had any impact on your parenting experience?

- Not at all
- A little
- A lot
- Not sure/don't know

If so how, please provide any detail:

* 30. Was the Cross Cultural Worker sensitive to your cultural needs and those of your family?

- ◯ Yes, always
- ◯ Yes, sometimes
- ◯ No
- ◯ Not sure/cannot remember

If you have any comments please provide:

* 31. How would you rate your level of satisfaction with the Cross Cultural Worker Service?

- ◯ Neither satisfied nor dissatisfied
- ◯ Satisfied
- ◯ Very satisfied
- ◯ Dissatisfied
- ◯ Very dissatisfied
- ◯ Not sure/don't know

If you have any comments please provide:

32. What did you like **most** about the Cross Cultural Worker Service?

33. What did you like **least** about Cross Cultural Worker Service?

34. If we were to improve the Cross Cultural Worker Service, what do you think we should change?

* 35. Would you recommend this service to your friends and family?

- ◯ Definitely won't
- ◯ Probably won't
- ◯ Maybe
- ◯ Probably will
- ◯ Definitely will
- ◯ Not sure/don't know

Please provide any additional comments you would like to make:

Please follow the link to provide name and telephone number if you would like to volunteer to be selected to also participate in a telephone conversation related to the Cross Cultural Workers in Maternity and Child & Family Health Service. The discussion would last about 30-25 minutes and give us more detail to help us improve our services. As stated earlier, your privacy will be protected.

Email link to volunteer for telephone conversation: ***SESLHD-CrossCulturalWorkers-Maternity&ChildAndFamilyHealth@health.nsw.gov.au***

Thank you for your time and participation!

English - Cross Cultural Workers in Maternity and Child & Family Health Services (12 month)

12 Month Postnatal Evaluation

Your experience of maternity and child and family health care is very important to us.

You have been invited to complete this survey because you have used the Cross Cultural Workers in Maternity and Child & Family Health Services. We are reviewing the Service and would like you to give us some feedback about your experience using the Service.

If you have already completed our survey in your pregnancy and/or when your baby was 6 months old, Thank You! Please complete this follow up survey too as it asks about your experience of the Cross Cultural Worker Service now your baby is 12 months old. Your feedback is very important as it helps us understand the quality of care you received and allows us to see where we need to improve.

Taking part in the survey is voluntary. We respect your decision to either complete or not complete the survey. If you decide not to participate, it will not affect the care and support you receive now or in the future. Whatever your decision, it will not affect your relationship with the staff caring for you.

Any information obtained will remain private and confidential. The survey will take about 10-15 minutes to complete.

Thank you in advance for completing the survey, we greatly appreciate your feedback to help us improve our Service.

Researcher:Helen Rogers - Early Parenting Program Coordinator
Child, Youth & Family Services
South Eastern Sydney Local Health District Health
Email: Helen.Rogers@health.nsw.gov.au

* 1. When did you first see the Cross Cultural Worker?

- ◯ During pregnancy
- ◯ After the birth of my baby

* 2. If you first saw the Cross Cultural Worker during pregnancy, have you already completed the pregnancy survey? (it would have been given to you at about 36 weeks of pregnancy).

- ◯ Yes
- ◯ No

* 3. Did you also complete a survey when your baby was approximately 6 months old?

- ◯ Yes
- ◯ No
- ◯ Cannot remember

* 4. What is the name of the country where you were born?

* 5. Did you arrive in Australia as a refugee or asylum seeker?

- Yes
- No
- Prefer not to answer

* 6. How long (years or months) have you lived in Australia?

7. Do you belong/identify with a cultural or ethnic group/s? If so, which one/s?

* 8. What is the name of the country where your partner was born?

Name of the country?

If born overseas, how long have they lived in Australia?

* 9. Which language do you mainly speak at home?

- English
- Other (please specify)

* 10. What is the highest level of education you have **completed?**

- ◯ Primary school
- ◯ Secondary to Year 12 or equivalent
- ◯ Technical college e.g. TAFE or equivalent
- ◯ University
- ◯ None of the above
- ◯ Other (please specify)

* 11. Is this your first baby?

- ◯ Yes
- ◯ No

12. If not your first baby, how many children do you have?

- ◯ 1
- ◯ 2
- ◯ 3
- ◯ 4 or more

13. What are the age(s) of your children (if applicable)?

* 14. How many weeks pregnant were you when you attended the hospital antenatal clinic in Australia for the **first time**?

- ◯ Less than 14 weeks pregnant
- ◯ 15- 19 weeks pregnant
- ◯ 20 - 24 weeks pregnant
- ◯ 25 - 28 weeks pregnant
- ◯ 29- 32 weeks pregnant
- ◯ 33 or more weeks pregnant
- ◯ I did not receive any antenatal care in Australia
- ◯ Not sure/cannot remember

* 15. How many weeks pregnant were you when you met the **Cross Cultural Worker** for the **first time**?

- Less than 14 weeks pregnant
- 15- 19 weeks pregnant
- 20 - 24 weeks pregnant
- 25 - 28 weeks pregnant
- 29- 32 weeks pregnant
- 33 or more weeks pregnant
- Baby less than 2 weeks old
- Baby between 2 weeks and 3 months old
- Baby older than 3 months
- Not sure/cannot remember

* 16. How old is your baby today?

* 17. How many weeks pregnant were you when you had your baby?

* 18. How many times did you talk with the Cross Cultural Worker **since your baby was 12 months old** (includes telephone, face to face, groups)?

- 1
- 2
- 3
- 4 or more
- Not sure/cannot remember

* 19. Have you seen **any** of the following for postnatal care for you and your baby? Please select all that apply.

- ☐ Not receiving any postnatal care
- ☐ Child and Family Health Nurse
- ☐ GP/Family Doctor
- ☐ Other, who

20. **If you are receiving postnatal care**, who is providing **most** of the care for you and your baby? Please select only one.

- ◯ Child and Family Health Nurse
- ◯ GP/Family Doctor
- ◯ Other, who

* 21. How prepared did you feel for becoming a parent?
1=Not prepared at all. 3=Neutral. 5=Well prepared. N/A=Not applicable

1	2	3	4	5
◯	◯	◯	◯	◯

* 22. Was it useful to have the Cross Cultural Worker talk to you and provide information in the previous 12 months?

- ◯ Yes
- ◯ No
- ◯ Not sure/cannot remember

If you have any comments please provide

* 23. Do you think the Cross Cultural Worker Service has supported you to understand information to prepare for parenting?

- Yes
- No
- Not applicable

If you have any comments please provide

* 24. Did you feel you could ask the Cross Cultural Worker any questions you had?

- No, never
- Yes, a few times
- Yes, most of the time
- Yes, all of the time
- Not sure/cannot remember

If you have any comments please provide

* 25. Did the Cross Cultural Worker Service meet your needs providing information on the following?

1=Not at all. 3=Neutral. 5=A lot. N/A=Not applicable

	1 Not at all	2	3 Neutral	4	5 A lot	N/A
Health services and facilities	○	○	○	○	○	○
Child and family health services	○	○	○	○	○	○
Where to find more information and support	○	○	○	○	○	○
How your partner could provide support	○	○	○	○	○	○
Child development	○	○	○	○	○	○
Entitlements to financial support, e.g. Centrelink, etc	○	○	○	○	○	○
Feeding your baby	○	○	○	○	○	○
Caring for your baby	○	○	○	○	○	○
Becoming a parent	○	○	○	○	○	○
Community supports, playgroups, services in your local area	○	○	○	○	○	○
Emotional support and support networks	○	○	○	○	○	○

* 26. In relation to question 24 overall, did you feel the Cross-Cultural Worker **communicated clearly** about the information she provided to you?

1=Not at all. 3=Neutral. 5=Very well. N/A=Not applicable

1 Not at all	2	3 Neutral	4	5 Very well	N/A
○	○	○	○	○	○

If you have any comments please provide:

* 27. Before you had your baby how would you describe your experience with babies less than 12 months old?

- ○ Lots of experience
- ○ Some experience
- ○ Very little experience
- ○ No experience

* 28. Which one word would best describe your thoughts about becoming a Mother **before** you met the Cross Cultural Worker?

- Very nervous
- Nervous
- OK/Neutral
- Excited
- Very excited
- I do not remember

* 29. Which one word would best describe your thoughts about becoming a Mother **after** you met the Cross Cultural Worker?

- Very nervous
- Nervous
- OK/Neutral
- Excited
- Very excited
- I do not remember

* 30. Do you think the Cross Cultural Worker Service had any impact on your parenting experience?

- Not at all
- A little
- A lot
- Not sure/don't know

If so how, please provide any detail:

* 31. Was the Cross Cultural Worker sensitive to your cultural needs and those of your family?

- ◯ Yes, always
- ◯ Yes, sometimes
- ◯ No
- ◯ Not sure/cannot remember

If you have any comments please provide:

* 32. How would you rate your level of satisfaction with the Cross Cultural Worker Service?

- ◯ Neither satisfied nor dissatisfied
- ◯ Satisfied
- ◯ Very satisfied
- ◯ Dissatisfied
- ◯ Very dissatisfied
- ◯ Not sure/don't know

If you have any comments please provide:

33. What did you like **most** about the Cross Cultural Worker Service?

34. What did you like **least** about Cross Cultural Worker Service?

35. If we were to improve the Cross Cultural Worker Service, what do you think we should change?

* 36. Would you recommend this service to your friends and family?

- ◯ Definitely won't
- ◯ Probably won't
- ◯ Maybe
- ◯ Probably will
- ◯ Definitely will
- ◯ Not sure/don’t know

Please add any additional comments you would like to make:

Thank you for your time and participation!

PARTNER Cross Cultural Workers in Maternity and Child & Family Health Services

Partner Evaluation 6 months

Your experience of maternity and child and family health care is very important to us.

You have been invited to complete this survey because you and your partner have used the Cross Cultural Workers in Maternity and Child & Family Health Services. We are reviewing the Service and would like you to give us some feedback about your experience using the Service.

Your experience is important as it helps us understand the quality of care you and your partner received and allows us to see where we need to improve. The survey asks about your experiences and satisfaction with the care you received.

Taking part in the survey is voluntary. We respect your decision to either complete or not complete the survey. If you decide not to participate, it will not affect the care and support you receive now or in the future. Whatever your decision, it will not affect your relationship with the staff caring for you.

Any information obtained will remain private and confidential. The survey will take about 10-15 minutes to complete. It will ask questions about you and your partner's experience using the Cross Cultural Worker Service.

Thank you in advance for completing the survey, we greatly appreciate your feedback to help us improve services provision.

Researcher: Helen Rogers - Early Parenting Program Coordinator
Child, Youth & Family Services
South Eastern Sydney Local Health District Health
Email: Helen.Rogers@health.nsw.gov.au

* 1. What is the name of the country where you were born?

____

* 2. If you were born overseas, did you arrive in Australia as a refugee or asylum seeker?

- ◯ Yes
- ◯ No
- ◯ Prefer not to answer

* 3. If you were born overseas, how long (years or months) have you lived in Australia?

____

* 4. Do you belong/identify with a cultural or ethnic group/s? If so, which one/s?

* 5. What is the name of the country where your partner was born?

Name of the country?

If born overseas, how long have they lived in Australia?

* 6. Which language do you mainly speak at home?

- English
- Other please specify

* 7. What is the highest level of education you have **completed?**

- Primary school
- Secondary to Year 12 or equivalent
- Technical college e.g. TAFE or equivalent
- University
- None of the above
- Other (please specify)

* 8. How old is your baby today?

* 9. Is this **your** first baby (even if your partner already has other children)?

- Yes
- No

10. If not your first baby, how many children do you have?

- 1
- 2
- 3
- 4 or more

11. What are the age(s) of your children (if applicable)?

* 12. How many weeks pregnant was your partner or how old was your baby when you met the Cross Cultural Worker for the **first time**?

- Less than 14 weeks pregnant
- 15- 19 weeks pregnant
- 20 - 24 weeks pregnant
- 25 - 28 weeks pregnant
- 29- 32 weeks pregnant
- 33 or more weeks pregnant
- I didn't attend any antenatal care with my partner
- Baby less than 2 weeks old
- Baby more than 2 weeks old
- Baby older than 3 months
- Baby older than 6 months
- Not sure/I cannot remember

* 13. How many times did you talk with the Cross Cultural Worker (includes telephone, face to face, groups)?

- 1
- 2
- 3
- 4 or more
- Not sure/I cannot remember
- I did not talk to the Cross Cultural Worker

* 14. How prepared did you feel for your partners' labour and birth?

1=Not prepared at all. 3=Neutral. 5=Well prepared.

1	2	3	4	5
○	○	○	○	○

* 15. Was it useful to have the Cross Cultural Worker talk to you and provide information and resources?

- ○ Yes
- ○ No
- ○ Not applicable -I do not remember seeing/talking to a Cross Cultural Worker

If you have any comments please provide

* 16. Did you feel the Cross Cultural Worker made you feel welcome and involved you in conversations?

- ○ Yes
- ○ No
- ○ Not applicable-I do not remember seeing a Cross Cultural Worker

If you have any comments please provide

* 17. Did you feel you could ask the Cross Cultural Worker any questions you had?

- ○ No, never
- ○ Yes, a few times
- ○ Yes, most of the time
- ○ Yes, all of the time
- ○ I do not remember seeing a Cross Cultural Worker

If you have any comments please provide

* 18. Did the Cross Cultural Worker Service meet your needs providing information on the following?

1=Not at all. 3=Neutral. 5=A lot. N/A=Not applicable

	1 Not at all	2	3 Neutral	4	5 A lot	N/A
Health services and facilities	○	○	○	○	○	○
Child and family health services	○	○	○	○	○	○
Antenatal and parenting education	○	○	○	○	○	○
Where to find more information and support	○	○	○	○	○	○
How to provide support to your partner	○	○	○	○	○	○
Child development	○	○	○	○	○	○
Entitlements to financial support, e.g. Centrelink, etc	○	○	○	○	○	○
Emotional changes in pregnancy and after the birth	○	○	○	○	○	○
hysical changes in pregnancy and after the birth	○	○	○	○	○	○
Feeding your baby	○	○	○	○	○	○
Caring for your baby	○	○	○	○	○	○
Becoming a parent	○	○	○	○	○	○
Community supports, playgroups, services in your local area	○	○	○	○	○	○
Emotional support and support networks	○	○	○	○	○	○

* 19. In relation to question 18 overall, did you feel the Cross-Cultural Worker **communicated clearly** about the information she provided to you?

1=Not at all. 3=Neutral. 5=Very well. N/A=Not applicable

1 Not at all	2	3 Neutral	4	5 Very well	N/A
○	○	○	○	○	○

If you have any comments please provide:

* 20. Do you think the Cross Cultural Worker had any impact on your partners' pregnancy and birth experience?

- ◯ None
- ◯ A little
- ◯ A lot
- ◯ Don't know

If you have any comments please provide:

[ ]

* 21. Before you had your baby how, would you describe your experience with a baby who was less than 6 weeks old?

- ◯ Lots of experience
- ◯ Some experience
- ◯ Very little experience
- ◯ No experience

* 22. Which one word would best describe your thoughts about becoming a parent **before** you met the Cross Cultural Worker?

- ◯ Very nervous
- ◯ Nervous
- ◯ OK/Neutral
- ◯ Excited
- ◯ Very excited
- ◯ Not sure/cannot remember
- ◯ Not applicable, I do not remember meeting the Cross Cultural Worker

* 23. Which one word would best describe your thoughts about becoming a parent **after** you met the Cross Cultural Worker?

- ◯ Very nervous
- ◯ Nervous
- ◯ OK/Neutral
- ◯ Excited
- ◯ Very excited
- ◯ Not sure/I cannot remember
- ◯ Not applicable, I do not remember meeting the Cross Cultural Worker

* 24. Do you think the Cross Cultural Worker Service had any impact on your parenting experience?

- ◯ None
- ◯ A little
- ◯ A lot
- ◯ Not sure/don't know
- ◯ Not applicable, I do not remember meeting the Cross Cultural Worker

If so have any comments please provide:

* 25. Was the Cross Cultural Worker sensitive to your cultural needs and those of your family?

- ◯ Yes, always
- ◯ Yes, sometimes
- ◯ No
- ◯ Not sure/cannot remember

If you have any comments please provide:

* 26. How would you rate your level of satisfaction with the Cross Cultural Worker Service?

- ◯ Neither satisfied nor dissatisfied
- ◯ Satisfied
- ◯ Very satisfied
- ◯ Dissatisfied
- ◯ Very dissatisfied
- ◯ Not sure/don't know

If you have any comments please provide:

27. What did you like **most** about the Cross Cultural Worker Service?

28. What did you like **least** about Cross Cultural Worker Service?

29. If we were to improve the Cross Cultural Worker Service, what do you think we should change?

* 30. Would you recommend this service to your friends and family?

- Definitely won't
- Probably won't
- Maybe
- Probably will
- Definitely will
- Not sure/don't know

Please provide any additional comments you would like to make:

Thank you for your time and participation!
