# Supplementary material for: Perspectives of women and partners from migrant and refugee backgrounds accessing the Cross Cultural Worker Service in maternity and early childhood services—a survey study
Source: BMC Health Serv Res. 2023 Nov 10;23:1233. doi: 10.1186/s12913-023-10194-3 (PMC10636916; doi:10.1186/s12913-023-10194-3)
Supplement: Supplementary file 2 — Additional file 2. [file 12913_2023_10194_MOESM2_ESM.docx]

**Additional file 2:**

**Perspectives of women and partners from migrant and refugee backgrounds accessing the Cross Cultural Worker Service in maternity and early childhood services - a survey study**

**Authors' list**

Helen J. Rogers* PhD Candidate, RM, RN, MPH ^1 2^

Professor Caroline SE Homer AO RM PhD ^3 4^

Associate Professor Amanda Henry PhD MPH FRANZCOG BMed BMedSci DDU (O&G) ^2 5 6^

**Author affiliations**

^1^ Child, Youth & Family Services, South Eastern Sydney Local Health District, Sydney, NSW, 2010, Australia

^2^ Discipline of Women’s Health, School of Clinical Medicine, University of NSW (UNSW), Sydney, NSW, 2000, Australia

^3^ Maternal and Child Health, Burnet Institute, Melbourne, Vic, 3004, Australia

^4^ Centre for Midwifery and Child and Family Health, Faculty of Health, University of Technology Sydney, NSW, 2007, Australia

^5^ Department of Women’s and Children’s Health, St George Hospital, Sydney, NSW, 2217, Australia

^6^ Australia Global Women’s Health Program, The George Institute for Global Health, Sydney, NSW, 2042, Australia

**Additional file 2:**

**Table S1 Pregnancy, 6-month, 12-month and partner 6-month postpartum surveys**

**Table S2 Thoughts about becoming a mother or parent**

**Table S3 Pregnancy: Cross Cultural Worker Service meeting needs on the following topics**

**Table S4 Maternal 6 month postnatal: Cross Cultural Worker Service meeting needs on the following topics**

**Table S5 Maternal 12 month postnatal: Cross Cultural Worker Service meeting needs on the following topics**

**Table S6 Partner 6 month: Cross Cultural Worker Service meeting needs on the following topics**

**Additional file 2**

**Table S1 Pregnancy, 6-month, 12-month and partner 6-month postpartum surveys**

| **Characteristic** | **Pregnancy**  **(n=113)**  **n (%)** | **6 months**  **(n=50)**  **n (%)** | **12 months (n=44)**  **n (%)** | **p value**  **(women)** | **Partner 6 months**  **(n=24)**  **(n) %** | **p value**  **(all participants)** |
| --- | --- | --- | --- | --- | --- | --- |
| **Country of Birth** | | | | **0.69** |  | **0.05** |
| Nepal | 53 (47) | 26 (52) | 26 (59) |  | 12 (50) |  |
| Indonesia | 20 (18) | 2 (4) | 5 (10) |  | 1 (4) |  |
| Bangladesh | 17 (15) | 5 (10) | 6 (14) |  | 3 (13) |  |
| India | 6 (5) | 4 (8) | 1 (2) |  | 2 (8) |  |
| Thailand | 6 (5) | 4 (0) | 3 (7) |  | 1 (4) |  |
| China | 2 (2) | 0 (0) | 0 (0) |  | 0 (0) |  |
| Mongolia | 2 (2) | 1 (2) | 0 (0) |  | 1 (4) |  |
| Philippines | 2 (2) | 1 (2) | 0 (0) |  | 0 (0) |  |
| Colombia | 1 (1) | 1 (2) | 2 (5) |  | 0 (0) |  |
| Peru | 1 (1) | 1 (2) | 1(2) |  | 0 (0) |  |
| Brazil | 1 (1) | 1 (2) | 0 (0) |  | 0 (0) |  |
| Chile | 1 (1) | 1 (2) | 0 (0) |  | 0 (0) |  |
| Spain | 1 (1) | 1 (2) | 0 (0) |  | 0 (0) |  |
| Pakistan | 0 (0) | 1 (2) | 0 (0) |  | 0 (0) |  |
| Kuwait | 0 (0) | 1 (2) | 0 (0) |  | 0 (0) |  |
| Taiwan | 0 (0) | 0 (0) | 0 (0) |  | 1 (4) |  |
| Australia | 0 (0) | 0 (0) | 0 (0) |  | 3 (13) |  |
| **Years in Australia** | | | | **0.06** |  | **<0.001** |
| ≤ 2 years | 26 (23) | 12 (24) | 7 (16) |  | 1 (4) |  |
| 3-5 years | 70 (62) | 28 (56) | 21 (47) |  | 4 (17) |  |
| ≥ 6 years | 17 (17) | 10 (20) | 16 (36) |  | 16 (67) |  |
| Australian born | 0 (0) | 0 (0) | 0 (0) |  | 3 (13) |  |
| **Identify as refugee or asylum seeker** | | | | **0.30** |  |  |
| Yes | 0 (0) | 0 (0) | 0 (0) |  | 0 (0) |  |
| No | 108 (96) | 49 (98) | 44 (100) |  | 0 (0) |  |
| Prefer not to say | 5 (4) | 1 (2) | 0 (0) |  | 0 (0) |  |
| **Highest level of education** | | | | **0.56** |  | **0.73** |
| Primary | 0 (0) | 1 (2) | 0 (0) |  | 0 (0) |  |
| ≤ Year 12 or equivalent | 12 (11) | 7 (14) | 6 (14) |  | 4 (17) |  |
| Technical college | 20 (18) | 4 (8) | 6 (14) |  | 5 (21) |  |
| University | 80 (71) | 38 (76) | 32 (73) |  | 15 (62) |  |
| Other | 1 (1) | 0 (0) | 0 (0) |  | 0 (0) |  |
| **Main language spoken at home** | | | | **0.65** |  | **0.38** |
| English | 13 (12) | 8 (16) | 7 (16) |  | 6 (25) |  |
| Other | 100 (89) | 42 (84) | 37 (84) |  | 18 (75) |  |
| **Parity** | | | | **0.39** |  | **0.59** |
| Primip | 104 (92) | 43 (86) | 41 (93) |  | 22 (92) |  |
| Multip | 9 (8) | 7 (14) | 3 (7) |  | 2 (8) |  |
| **Gestation 1st hospital antenatal appointment in Australia*** | | | | **0.77** |  |  |
| ≤14 weeks | 34 (30) | 14 (28) | 9 (21) |  |  |  |
| 15 - 19 weeks | 49 (43) | 20 (40) | 25 (57) |  |  |  |
| 20 - 24 weeks | 21 (19) | 13 (26) | 5 (12) |  |  |  |
| 25 - 28 weeks | 4 (4) | 1 (2) | 1 (2) |  |  |  |
| 29 - 32 weeks | 2 (2) | 1 (2) | 2 (5) |  |  |  |
| ≥33 weeks | 1 (1) | 0 (0) | 1 (2) |  |  |  |
| Not sure/cannot remember | 2 (2) | 1 (2) | 1 (2) |  |  |  |
| **Gestation 1st CCW meeting** | | | | **0.12** |  | **0.02** |
| ≤14 weeks | 11 (10) | 7 (14) | 3 (7) |  | 1 (4) |  |
| 15 - 19 weeks | 32 (28) | 17 (34) | 20 (46) |  | 8 (33) |  |
| 20 - 24 weeks | 30 (27) | 13 (26) | 10 (23) |  | 7 (30) |  |
| 25 - 28 weeks | 14 (12) | 11 (22) | 4 (9) |  | 1 (4) |  |
| 29 - 32 weeks | 11 (10) | 0 (0) | 3 (7) |  | 0 (0) |  |
| ≥33 weeks | 4 (4) | 2 (4) | 2 (5) |  | 1 (4) |  |
| Baby <2 weeks old | 0 (0) | 0 (0) | 0 (0) |  | 1 (4) |  |
| Not sure/cannot remember | 11 (10) | 0 (0) | 2 (5) | 43 | 5 (21) |  |
| **Number of CCW contacts** | | | | **<0.001** |  | **<0.001** |
| 1 | 2 (2) | 12 (24) | 7 (16) |  | 2 (8) |  |
| 2 | 8 (7) | 6 (12) | 4 (9) |  | 2 (8) |  |
| 3 | 41 (36) | 18 (36) | 21 (48) |  | 7 (29) |  |
| 4 or more | 62 (55) | 14 (28) | 12 (27) |  | 8 (33) |  |
| Did not talk to the CCW | 0 (0) | 0 (0) | 0 (0) |  | 5 (21) |  |
| **Was it useful to have the CCW talk to you and provide information/resources?** | | | | **0.01** |  | **0.02** |
| No | 2 (2) | 2 (4) | 1 (2) |  | 0 (0) |  |
| Yes | 111 (98) | 42 (84) | 38 (86) |  | 21 (88) |  |
| Not sure/cannot remember | 0 (0) | 6 (12) | 5 (11) |  | 3 (12)  N/A Did not talk to CCW |  |
| **Did the CCW make you feel welcome and involved in conversations? **** | | | | | | **N/A** |
| Yes |  |  |  |  | 19 (79) |  |
| N/A-do not remember seeing CCW |  |  |  |  | 5 (21) |  |
| **Supported understanding information to prepare for pregnancy, birth, parenting (AN) or parenting (PN)** | | | | **0.96** |  | **0.71** |
| Yes | 113 (100) | 48 (96) | 42 (95) |  | 22 (95) |  |
| No | 0 (0) | 1 (2) | 2 (5) |  | 2 (5) |  |
| N/A | 0 (0) | 1 (2) | 0 (0) |  | 0 (0) |  |
| **Could you ask the CCW any questions you had** | | | | **0.05** |  | **<0.001** |
| Yes, a few times | 20 (18) | 13 (26) | 10 (23) |  | 5 (21) |  |
| Yes, most of the time | 34 (30) | 14 (28) | 15 (34) |  | 6 (25) |  |
| Yes, all of the time | 56 (50) | 21 (42) | 13 (30) |  | 11 (46) |  |
| Not sure/don't know | 1 (1) | 1 (2) | 4 (9) |  | 0 (0) |  |
| No, never | 0 (0) | 1 (2) | 2 (5) |  | 2 (8) Do not remember seeing CCW |  |
| Missing | 2 (2) | 0 (0) | 0 (0) |  | 0 (0) |  |
| **Impact on your partners’ pregnancy and birth experience**** | | | |  |  | **N/A** |
| Not at all |  |  |  |  | 1 (4) |  |
| A little |  |  |  |  | 2 (8) |  |
| A lot |  |  |  |  | 19 (79) |  |
| Not sure/don't know |  |  |  |  | 2 (8) |  |
| **Impact on pregnancy (AN) or parenting (PN) experience** | | | | **0.28** |  | **0.12** |
| Not at all | 1 (1) | 3 (6) | 3 (7) |  | 0 (0) |  |
| A little | 23 (20) | 12 (24) | 7 (16) |  | 6 (25) |  |
| A lot | 85 (76) | 32 (64) | 30 (68) |  | 17 (71) |  |
| Not sure/don't know | 3 (3) | 3 (6) | 4 (9) |  | 1 (4) Do not remember seeing CCW |  |
| Missing | 1 (1) | 0 (0) | 0 (0) |  | 0 (0) | 0 (0) |
| **Sensitive to your cultural needs and those of your family** | | | | **0.36** |  | **0.40** |
| Not sure/cannot remember | 11 (10) | 4 (8) | 6 (14) |  | 5 (21) |  |
| No | 3 (3) | 6 (12) | 3 (7) |  | 2 (8) |  |
| Yes, sometimes | 24 (21) | 8 (16) | 8 (18) |  | 3 (13) |  |
| Yes, always | 73 (65) | 32 (64) | 27 (61) |  | 14 (58) |  |
| Missing | 2 (2) | 0 (0) | 0 (0) |  | 0 (0) |  |
| **Level of satisfaction** | | | | **0.29** |  | **0.42** |
| Very dissatisfied | 3 (3) | 2 (4) | 0 (0) |  | 0 (0) |  |
| Dissatisfied | 1 (1) | 0 (0) | 1 (2) |  | 0 (0) |  |
| Neither satisfied/dissatisfied | 3 (3) | 4 (8) | 0 (0) |  | 1 (4) |  |
| Satisfied | 43 (38) | 17 (34) | 19 (43) |  | 7 (29) |  |
| Very satisfied | 63 (56) | 27 (54) | 23 (52) |  | 15 (63) |  |
| Not sure/don’t know | 0 (0) | 0 (0) | 1 (2) |  | 1 (4) |  |
| **Recommend CCW Service to friends and family** | | | | **0.15** |  | **0.04** |
| Definitely won’t | 0 (0) | 0 (0) | 1 (2) |  | 1 (4) |  |
| Maybe | 3 (3) | 4 (8) | 0 (0) |  | 0 (0) |  |
| Probably will | 16 (14) | 9 (18) | 8 (18) |  | 2 (8) |  |
| Definitely will | 94 (83) | 37 (74) | 35 (80) |  | 20 (83) |  |
| Not sure/don’t know | 0 (0) | 0 (0) | 0 (0) |  | 1 (4) |  |

**Table S2 Thoughts about becoming a mother or parent**

| **Pregnancy** | | | | **6 months** | | | **12 months** | | | **p value**  **(women participants)** | **Partner 6 months** | | | **p value**  **(All participants)** |
| --- | --- | --- | --- | --- | --- | --- | --- | --- | --- | --- | --- | --- | --- | --- |
| **Thoughts about becoming a mother/parent** | **Before meeting CCW** | **After** **meeting CCW** | **P value** | **Before meeting CCW** | **After** **meeting CCW** | **P value** | **Before meeting CCW** | **After** **meeting CCW** | **P value** |  | **Before meeting CCW** | **After** **meeting CCW** | **p value** |  |
|  | **n (%)** | **n (%)** | **<0.001** | **n (%)** | **n (%)** | **0.015** | **n (%)** | **n (%)** | **0.06** | **<0.001** | **n (%)** | **n (%)** | **0.45** | **0.12** |
| Very nervous | 15 (13) | 1 (1) |  | 1 (2) | 0 (0) |  | 3 (7) | 0 (0) |  |  | 1 (4) | 0 (0) |  |  |
| Nervous | 22 (20) | 2 (2) |  | 10 (20) | 1 (2) |  | 8 (18) | 3 (7) |  |  | 1 (4) | 0 (0) |  |  |
| OK/Neutral | 23 (20) | 23 (20) |  | 11 (22) | 13 (26) |  | 9 (21) | 7 (16) |  |  | 7 (29) | 4 (17) |  |  |
| Excited | 39 (35) | 52 (46) |  | 17 (34) | 16 (32) |  | 19 (43) | 19 (43) |  |  | 10 (42) | 9 (38) |  |  |
| Very excited | 10 (9) | 33 (29) |  | 8 (16) | 19 (38) |  | 5 (11) | 14 (32) |  |  | 4 (17) | 9 (38) |  |  |
| Not sure/don’t know | 4 (4) | 2 (2) |  | 3 (6) | 1 (2) |  | 0 (0) | 1 (2) |  |  | 0 (0) | 1 (4) |  |  |
| Do not remember meeting CCW | 0 (0) | 0 (0) |  | 0 (0) | 0 (0) |  | 0 (0) | 0 (0) |  |  | 1 (4) | 1 (4) |  |  |
| **Total (%)** | **113 (100)** | **113 (100)** |  | **50**  **(100)** | **50**  **(100)** |  | **44**  **(100)** | **44**  **(100)** |  |  | **24**  **(100)** | **24**  **(100)** |  |  |

**Table S3 Pregnancy: Cross Cultural Worker Service meeting needs on the following topics**

| **Did the CCW Service meet your needs providing information on…**  **n=113** | **Health services & facilities** | **Child and family health services** | **Antenatal and parenting education** | **Where to find more information and support** | **How your partner could provide support** | **Child development** | **Financial**  **Entitlements**  **/Support** | **Emotional changes in pregnancy and after the birth** | **Physical changes in pregnancy and after the birth** | **Feeding your baby** | **Caring for your baby** | **Becoming a parent** | **Community supports, playgroups, services in local area** | **Emotional support and support networks** | **Did the CCW clearly communicate information** |
| --- | --- | --- | --- | --- | --- | --- | --- | --- | --- | --- | --- | --- | --- | --- | --- |
|  | **n %** | **n %** | **n %** | **n %** | **n %** | **n %** | **n %** | **n %** | **n %** | **n %** | **n %** | **n %** | **n %** | **n %** | **n (%)** |
| 1=Not at all | 0 (0) | 0 (0) | 0 (0) | 0 (0) | 0 (0) | 2 (2) | 8 (7) | 1 (1) | 0 (0) | 0 (0) | 1 (1) | 1 (0.9) | 5 (4) | 2(2) | 0 (0) |
| 2 | 0 (0) | 1(1) | 0 (0) | 0 (0) | 0 (0) | 0 (0) | 4 (4) | 0 (0) | 0 (0) | 0 (0) | 0 (0) | 0 (0) | 1 (1) | 0 (0) | 0 (0) |
| 3=Neutral | 16 (14) | 14 (12) | 12 (11) | 12 (11) | 19 (17) | 20 (18) | 30 (27) | 18 (16) | 25 (22) | 14 (12) | 20 (18) | 16 (14) | 30 (27) | 18 (16) | 10 (9) |
| 4 | 17(15) | 16 (14) | 13 (12) | 13 (12) | 18 (16) | 20 (18) | 20 (18) | 16 (14) | 19 (17) | 15 (13) | 14 (12) | 17 (15) | 12 (11) | 17(15) | 7 (6) |
| 5= A lot | 78 (69) | 77 (68) | 87 (77) | 87 (77) | 74 (66) | 4 (4) | 41 (36) | 74 (66) | 66 (58) | 82(73) | 77(68) | 75 (66) | 57 (50) | 73 (65) | 96 (85)  very well |
| N/A | 1(0.9) | 3 (3) | 0 (0) | 0 (0) | 0 (0) | 4(4) | 6 (5) | 3 (3) | 1 (1) | 1 (1) | 0 (0) | 1 (1) | 5 (4) | 1 (1) | 0 (0) |
| **Total** | 0 (0) | **111 (98)** | **112 (99)** | **112 (99)** | **111 (98)** | **110 (97)** | **109 (97)** | **112 (99)** | **111 (98)** | **112 (99)** | **112 (99)** | **110 (97)** | **110 (97)** | **111 (98)** | **113 (100)** |
| **Median**  **(IQR)** | 5 (4-5) | 5 (4-5) | 5 (5) | 5(5) | 5 (4-5) | 5 (4-5) | 4 (3-5) | 5 (4-5) | 5 (4-5) | 5 (4-5) | 5 (4-5) | 5 (4-5) | 5 (3-5) | 5 (4-5) | 5 (5) |
| **Mean (SD)** | 4.6 (0.7) | 4.6 (0.8) | 4.7 (0.7) | 4.7 (0.7) | 4.5 (0.8) | 4.4 (0.9) | 3. 9 (1.3) | 4.5 (0.9) | 4.4 (0.8) | 4.6 (0.7) | 4.5 (0.8) | 4.5 (0.8) | 4.2 (1.2) | 4 (0.9) | 4.8 (0.6) |

**Table S4 Maternal 6 month postnatal: Cross Cultural Worker Service meeting needs on the following topics**

| **Did the CCW Service meet your needs providing information on…**  **n=50** | **Health services & facilities** | **Child and family health services** | **Where to find more information and support** | **How your partner could provide support** | **Child development** | **Entitlements to financial support** | **Feeding your baby** | **Caring for your baby** | **Becoming a parent** | **Community supports, playgroups, services in your local area** | **Emotional support and support networks** | **Did the CCW clearly communicate information** |
| --- | --- | --- | --- | --- | --- | --- | --- | --- | --- | --- | --- | --- |
|  | **n (%)** | **n (%)** | **n (%)** | **n (%)** | **n (%)** | **n (%)** | **n (%)** | **n (%)** | **n (%)** | **n (%)** | **n (%)** | **n (%)** |
| 1=Not at all | 0 (0) | 2 (4) | 3 (6) | 0 (0) | 2 (4) | 8 (16) | 1 (2) | 2 (4) | 1 (2) | 4 (8) | 1 (2) | 0 (0) |
| 2 | 0 (0) | 1 (2) | 0 (0) | 1 (2) | 2 (4) | 7 (14) | 0 (0) | 0 (0) | 0 (0) | 3 (6) | 4 (8) | 1 (2) |
| 3=Neutral | 4 (8) | 1 (2) | 3 (6) | 6 (12) | 6 (12) | 9 (18) | 10 (20) | 10 (20) | 13 (26) | 15 (30) | 11 (2) | 9 (18) |
| 4 | 14 (28) | 14 (28) | 13 (26) | 10 (20) | 12 (24) | 12 (24) | 11 (22) | 14 (28) | 10 (20) | 10 (20) | 13 (26) | 11 (22) |
| 5= A lot | 32 (64) | 32 (64) | 31 (62) | 33 (66) | 28 (56) | 10 (20) | 28 (56) | 24 (48) | 26 (52) | 18 (36) | 21 (42) | 28 (56)  =very well |
| Missing | 0 (0) | 0 (0) | 0 (0) | 0 (0) | 0 (0) | 4 (8) | 0 (0) | 0 (0) | 0 (0) | 0 (0) | 0 (0) | 1 (2) |
| **Total** | **50 (100)** | **50 (100)** | **50 (100)** | **50 (100)** | **50 (100)** | **50 (100)** | **50 (100)** | **50 (100)** | **50 (100)** | **50 (100)** | **50 (100)** | **50 (100)** |
| **Median (IQR)** | 5 (4-5) | 5 (4-5) | 5 (4-5) | 5 (4-5) | 5 (4-5) | 4 (2-5) | 5 (4-5) | 4 (3.8-5) | 5 (3-5) | 4 (3-5) | 4 (3-5) | 5 (4-5) |
| **Mean (SD)** | 4.6 (0.6) | 4.5(0.9) | 4.4(1.0) | 4.5(0.8) | 4.2(1.1) | 3.4(1.6) | 4.3(0.9) | 4.2(1.0) | 4.2(0.9) | 3.7 (1.2) | 4(1.1) | 4.4 (0.9) |

**Table S5 Maternal 12 month postnatal: Cross Cultural Worker Service meeting needs on the following topics**

| **Did the Cross Cultural Worker Service meet your needs providing information on…**  **n=44** | **Health services & facilities** | **Child and family health services** | **Where to find more information and support** | **How your partner could provide support** | **Child development** | **Entitlements to financial support** | **Feeding your baby** | **Caring for your baby** | **Becoming a parent** | **Community supports, playgroups, services in your local area** | **Emotional support and support networks** | **Did the CCW clearly communicate information** |
| --- | --- | --- | --- | --- | --- | --- | --- | --- | --- | --- | --- | --- |
|  | **n (%)** | **n (%)** | **n (%)** | **n (%)** | **n (%)** | **n (%)** | **n (%)** | **n (%)** | **n (%)** | **n (%)** | **n (%)** | **n (%)** |
| 1=Not at all | 1 (2) | 1 (2) | 0 (0) | 0 (0) | 1 (2) | 6 (14) | 2 (5) | 1 (2) | 0 (0) | 3 (7) | 0 (0) | 0 (0) |
| 2 |  | 1 (2) | 0 (0) | 1 (2) | 0 (0) | 5(11) | 0 (0) | 1 (2) | 0 (0) | 3 (7) | 1 (2) | 0 (0) |
| 3=Neutral | 7 (16) | 9 (21) | 7 (16) | 8 (18) | 10 (23) | 12 (27) | 16 (36) | 10 (23) | 11 (25) | 15 (34) | 12 (27) | 6 (14) |
| 4 | 8 (18) | 9 (21) | 8 (18) | 4 (9) | 5 (11) | 3 (7) | 7 (16) | 9 (21) | 11 (25) | 7 (16) | 10 (23) | 6 (14) |
| 5= A lot | 27 (61) | 24 (55) | 26 (59) | 30 (68) | 26 (59) | 7 (16) | 18 (41) | 22 (50) | 21 (48) | 13 (30) | 20 (46) | 31 (71)  = very well |
| N/A | 1 (2) | 0 (0) | 3 (7) | 1 (2) | 2 (5) | 11 (25) | 1 (2) | 1 (2) | 1 (2) | 3 (7) | 1 (2) | 0 (0) |
| Missing | 0 (0) | 0 (0) | 0 (0) | 0 (0) | 0 (0) | 0 (0) | 0 (0) | 0 (0) | 0 (0) | 0 (0) | 0 (0) | 1 (2) |
| **Total** | **44 (100)** | **44 (100)** | **44 (100)** | **44 (100)** | **44 (100)** | **44 (100)** | **44 (100)** | **44 (100)** | **44 (100)** | **44 (100)** | **44 (100)** | **44 (100)** |
| **Median (IQR)** | 5 (4-5) | 5 (3.3-5) | 5 (4-5) | 5 (4-5) | 5 (3.3-5) | 3 (2.3-5) | 4 (3-5) | 5 (3-5) | 4.5(3.3-5) | 5 (3-5) | 4 (3-5) | 5 (4-5) |
| **Mean (SD)** | 4.4 (1.0) | 4.2 (1.0) | 4.6 (0.8) | 4.5 (0.9) | 4.4 (1.0) | 3.8 (1.8) | 4 (1.1) | 4.2 (1.0) | 4.3 (0.9) | 3.8 (1.3) | 4.2 (0.9) | 4.5 (1.0) |

|  |
| --- |

**Table S6 Partner 6 month: Cross Cultural Worker Service meeting needs on the following topics**

| **Did the Cross Cultural Worker Service meet your needs providing information on…**  **n=24** | **Health services & facilities** | **Child and family health services** | **Where to find more information and support** | **Antenatal education and support** | **How to provide support to your partner** | **Child development** | **Entitlements to financial support** | **Emotional changes in pregnancy and after birth** | **Physical changes in pregnancy and after the birth** | **Feeding your baby** | **Caring for your baby** | **Becoming a parent** | **Community supports, playgroups, services in your local area** | **Emotional support and support networks** | **Did the CCW clearly communicate information** |
| --- | --- | --- | --- | --- | --- | --- | --- | --- | --- | --- | --- | --- | --- | --- | --- |
|  | n (%) | n (%) | n (%) | n (%) | n (%) | n (%) | n (%) | n (%) | n (%) | n (%) | n (%) | n (%) | n (%) | n (%) | n (%) |
| 1=Not at all | 0 (0) | 0 (0) | 0 (0) | 0 (0) | 0 (0) | 0 (0) | 2 (8) | 0 (0) | 0 (0) | 0 (0) | 0 (0) | 1 (4) | 0 (0) | 0 (0) | 0 (0) |
| 2 | 0 (0) | 0 (0) | 0 (0) | 0 (0) | 0 (0) | 0 (0) | 2 (8) | 2 (8) | 0 (0) | 0 (0) | 0 (0) | 0 (0) | 0 (0) | 0 (0) | 0 (0) |
| 3=Neutral | 4 (17) | 4 (17) | 6 (25) | 5 (21) | 3 (13) | 5 (21) | 8 (33) | 5 (21) | 9 (38) | 6 (25) | 6 (25) | 7 (29) | 10 (42) | 5 (21) | 5 (21) |
| 4 | 4 (17) | 2 (8) | 3 (13) | 2 (8) | 2 (8) | 4 (17) | 3 (13) | 2 (8) | 2 (8) | 4 (17) | 4 (17) | 3 (13) | 1 (4) | 7 (29) | 7 (29) |
| 5= A lot | 15 (63) | 17 (71) | 14 (58) | 16 (67) | 18 (75) | 14 (58) | 8 (33) | 14 (58) | 12 (50) | 13 (54) | 13 (54) | 12 (50) | 12 (50) | 11 (46) | 11 (46)  = very well |
| N/A | 1 (4) | 1 (4) | 1 (4) | 1 (4) | 1 (4) | 1 (4) | 1 (4) | 1 (4) | 1 (4) | 1 (4) | 1 (4) | 1 (4) | 1 (4) | 1 (4) | 0 (0) |
| Missing | 0 (0) | 0 (0) | 0 (0) | 0 (0) | 0 (0) | 0 (0) | 0 (0) | 0 (0) | 0 (0) | 0 (0) | 0 (0) | 0 (0) | 0 (0) | 0 (0) | 1 (4) |
| **Total** | **24(100)** | **24(100)** | **24(100)** | **24(100)** | **24(100)** | **24(100)** | **24(100)** | **24(100)** | **24(100)** | **24(100)** | **24(100)** | **24(100)** | **24(100)** | **24(100)** | **24(100)** |
| **Median (IQR)** | 5 (4-5) | 5(4.25-5) | 5 (4-5) | 5 (3.25-5) | 5 (5) | 5 (4-5) | 3.5 (3-5) | 5 (3-5) | 5 (3-5) | 5(3.25-5) | 5(3.25-5) | 5 (3-5) | 5 (3-5) | 4.5 (4-5) | 4 (3.25-5) |
| **Mean (SD)** | 4.5 (0.8) | 4.6 (0.8) | 4.5 (0.9) | 4.4 (0.9) | 4.7 (0.7) | 4.4 (0.9) | 3.7 (1.4) | 4.3 (1.1) | 4.2 (1.0) | 4.4 (0.9) | 4.4 (0.9) | 4.4 (0.9) | 4.2 (1.0) | 4.3 (0.9) | 4.1 (1.2) |

|  |
| --- |
