# Supplementary material for: Perspectives of women and partners from migrant and refugee backgrounds accessing the Cross Cultural Worker Service in maternity and early childhood services—a survey study
Source: BMC Health Serv Res. 2023 Nov 10;23:1233. doi: 10.1186/s12913-023-10194-3 (PMC10636916; doi:10.1186/s12913-023-10194-3)
Supplement: Supplementary file 3 — Additional file 3. [file 12913_2023_10194_MOESM3_ESM.docx]

**Additional file 3: Select quotes from free text survey responses**

**Perspectives of women and partners from migrant and refugee backgrounds accessing the Cross Cultural Worker Service in maternity and early childhood services - a survey study**

**Authors' list**

Helen J. Rogers* PhD Candidate, RM, RN, MPH ^1 2^

Professor Caroline SE Homer AO RM PhD ^3 4^

Associate Professor Amanda Henry PhD MPH FRANZCOG BMed BMedSci DDU (O&G) ^2 5 6^

**Author affiliations**

^1^ Child, Youth & Family Services, South Eastern Sydney Local Health District, Sydney, NSW, 2010, Australia

^2^ Discipline of Women’s Health, School of Clinical Medicine, University of NSW (UNSW), Sydney, NSW, 2000, Australia

^3^ Maternal and Child Health, Burnet Institute, Melbourne, Vic, 3004, Australia

^4^ Centre for Midwifery and Child and Family Health, Faculty of Health, University of Technology Sydney, NSW, 2007, Australia

^5^ Department of Women’s and Children’s Health, St George Hospital, Sydney, NSW, 2217, Australia

^6^ Australia Global Women’s Health Program, The George Institute for Global Health, Sydney, NSW, 2042, Australia

**Pregnancy survey select quotes**

**Liked most about CCW Service:**

- *A lot of BAD information everywhere, she can give me accurate information for all the process.*
- *It gives an opportunity to talk and share the issues we have and feel easier talking on your native language.*
- *The talk is very useful for my emotional wellbeing.*
- *They are excellent, friendly, helpful. The information and resources were good. It helps us to prepare physically, mentally.*
- *They explained me in details that I could not understand by a doctor visit.*
- *CCW Service really supports me to understand information to prepare for pregnancy, birth and parenting. Because it is my first pregnancy everything is new for me, service, has taught me lots of thing about pregnancy. Although because of COVID-19 we had only 4 class, it is really helpful.*
- *It helps me a lot. I shared all the information with my husband as well, we are very happy. Thank you for providing such an informative session. It helps us to prepare for pregnancy, birth, and parenting. All the staff are very friendly and helpful. Clear all our queries and concerns.*
- *Absolutely this is the most important service and Mom needs to carry out a good pregnancy.*
- *She was really good with her job; she knew what she was doing. It really helped me. We both are from same country so it was easy to communicate.*
- *Before I didn't have knowledge regarding pregnancy, birth, and parenting but now I have knowledge regarding pregnancy, birth, and parenting which makes me confident, and to cope with all these changes smoothly.*
- *After CCW program I came to know the best thing pregnant women can do to maintain right health during pregnancy, how to ger easier labour, how to raise and nurture a baby with best care.*
- *They had good knowledge of South Asian women and South Asian culture.*
- *We could put forward our queries without any second thought. CCW service made me feel comfortable and had less hesitant if I had any concerns regarding my pregnancy.*
- *Very helpful, felt emotionally supported, prepared us mentally for the journey.*
- *They were very informative and I was comfortable to ask them anything.*
- *They are kind, friendly, helpful, and always there whenever needed.*
- *I feel so good to know that CCW service are there for us in this foreign country to support for those who need help.*
- *I can talk with her in my own language and sharing my pregnancy experience.*
- *Detailed information about the support needs during pregnancy and after birth, opportunity to come up openly without hesitation. Also feel very secure and equally important being a cross culture living in Australia, and very thankful to this service.*
- *As a student I have financial difficulties, she helped me to get a car seat, cot and pram for free.*
- *CCW made my pregnancy much more calm and confident.*

**Liked least about CCW Service:**

- *The hours in person at the hospital.*
- *Need more sessions regarding the postnatal depression for both father and mother and basically more about accessing Centrelink and social support for the families rather than providing a brochure.*

**Recommendations for CCW Service improvement**

- *More CCW from different ethnic groups needed.*
- *Maybe you should start allowing partners to the group.*
- *Ensuring more workers.*
- *Don't need to improve. Everything perfect and better than the doctor. I am very happy to visit the hospital when I meet the CCW.*

**Maternal 6 month postnatal select quotes**

**Liked most about CCW Service:**

- *It was very helpful to have someone who can speak your language. It was more easy for me to talk and rely my words during those time. Since English isn’t my primary language, being able to meet cross Cultural Worker… who speaks Nepali was very helpful. I am really grateful for their support, information during my pregnancy.*
- *Very clear, a nice and friendly person. She makes me feel safe and I always received support.*
- *I felt very confident to asking about everything that I wasn’t sure how to do.*
- *Having cross worker with same background help us to speak on same language.*
- *Sincerely when you are a mom, in a country that is not yours, you require a lot of information about how the health system works and what kind of state support network exists.*
- *They made me confident. After my baby was born, I learnt a lot but most of them I already knew from the parenting class so I knew what to do. The way they guided us was awesome.*
- *They would listen* *to our concerns and try to reach us via phone or email ... they provided me with the baby bundle as well, which was very helpful since I was an overseas international student at the moment ... really helpful and appreciated a lot.*

**Liked least about CCW Service:**

- *I can't remember them giving any info about Centrelink and benefits.*
- *Do not give all the information available, I think that I still do not know who to turn to in many cases, especially with my post delivery process and my baby development.*
- *Haven’t had much support after having my baby.*

**Recommendations for CCW Service improvement**

- *I think we should get to meet them after birth so we can learn how to look after baby and ourselves.*
- *Mainly you should understand that people do not know how the health service works, so the more information given on all areas involved, it will be better, it will depend on each family to take or not this information.*
- *I wish they allow partners as well so mothers will get more mental support from their husband/partner.*
- *To improve CCW I think there should be more volunteers* *and more lessons. Like how to be dad class, how they can help their partners during and after pregnancy.*

**Maternal 12 month postnatal select quotes**

**Liked most about CCW Service:**

- *CCW is very helpful and can help us to connect and understand more about how the medical system work in this country.*
- *Very helpful with lots of information and emotional support as well.*
- *It’s very useful for me. Cause I am a bit nervous and stressful, but the CCW explain for me everything and that makes me feel better than before. Cause I am too worried about everything. And I am feeling more confident to tell everything to the Cross Cultural, more than my midwife. She is very very friendly! I am so happy that I met her!*
- *She always supported me, even when I am giving birth, she always texts me and ask about my condition. She gave me so many advice!*
- *Helpful in providing information regarding community health service/nurse, sharing experiences so I could relate.*
- *They gave us good confidence, mentally without any tension for having a happy labour by explaining all the possible things may happen.*
- *They give us so much knowledge about childbirth to me and my husband...it was very informative.*
- *CCW provided a lot of information and support. Also, it is good to know that’s there is someone is available for us to reach out if we need any help.*
- *Getting linked with other mothers’ group, emotional support. Explaining every little thing. All I want to say to CCWS is a big thank you.*
- *It's good to see other mother from same culture in similar phase and some new mums have become a great friend.*
- *The information provided by them played important role for us to look after the baby.*

**Liked least about CCW Service:**

- *Maybe because my pregnancy during COVID, so we meet less face to face.*
- *Partner should be allowed to the parenting class. It’s very important for a mum to have support from her partner. Without attending parenting class it’s very hard.*

**Recommendations for CCW Service improvement**

- *Give more information regarding postpartum as well so makes you more confident.*
- *Having at least a month meeting after baby is born, contacting mothers as we feel lost sometimes.*
- *Home visit after childbirth would be more helpful.*
- *I think they should know more about Centrelink stuff.*
- *I think we need more support like CCW. Please hire one more person with Indonesia background. because CCW could not come every day.*
- *More focus and information about postnatal checkups and information about benefit by Centrelink.*

**PARTNER 6-month survey select quotes**

**Liked most about CCW Service:**

- *For people like us coming to different country with different culture, CCW really helps. Easy to communicate and easy to learn.*
- *It was useful to make sure that no information was misunderstood. The worker with the same culture definitely understands how to calm first time mom.*
- *Helped make my wife feel very comfortable about accessing available resources in what was otherwise a foreign environment having only arrived in Australia last year and where English is not her first language.*

**Liked least about CCW Service:**

- *Due to COVID-19 situation I can’t really meet them much, but my wife used to tell me what sort of support I should give her but still it would be more useful if I can attend physically.*

**Recommendations for CCW Service improvement**

- *From my experience I feel like I should have been there with my wife when she was doing parenting classes so I would have got some detailed idea what is going on with her mind/body. How I can make/feel her comfortable.*
- *The information was way more helpful. It would be more great if authority allows partners at the parenting classes so I could have known more about what my wife will go through & how i can help her more.*
